# Down-regulation of neuroprotective protein kinase D in Huntington´s disease

**DOI:** 10.1038/s41419-025-07688-9

**Published:** 2025-06-03

**Authors:** Álvaro Sebastián-Serrano, Ana Simón-García, María Santos-Galindo, Marina Prudencio Sánchez-Carralero, Alberto H.-Alcántara, Cristina Clemente, Julia Pose-Utrilla, Miguel R. Campanero, Eva Porlan, José J. Lucas, Teresa Iglesias

**Affiliations:** 1 Instituto de Investigaciones Biomédicas Sols-Morreale. Consejo Superior de Investigaciones Científicas-Universidad Autónoma de Madrid (CSIC-UAM), Madrid, Spain; 2 Centro de Investigación Biomédica en Red de Enfermedades Neurodegenerativas (CIBERNED), Instituto de Salud Carlos III, Madrid, Spain; 3 Department of Biochemistry and Molecular Biology, Medical School, Complutense University of Madrid, Madrid, Spain; 4 Molecular Neuropathology Unit, Centro de Biología Molecular Severo Ochoa (CSIC-UAM), Madrid, Spain; 5 Tissue and Organ Homeostasis Program, Centro de Biología Molecular Severo Ochoa (CSIC-UAM), Madrid, Spain; 6 Centro de Investigación Biomédica en Red de Enfermedades Cardiovasculares (CIBERCV), Instituto de Salud Carlos III, Madrid, Spain; 7 Departamento de Biología Molecular, Universidad Autónoma de Madrid, C/ Francisco Tomás y Valiente, 7, Ciudad Universitaria de Cantoblanco, Madrid, Spain; 8 Instituto Universitario de Biología Molecular – UAM, Madrid, Spain; 9 Instituto de Investigación Sanitaria del Hospital Universitario La Paz (IdiPAZ), Instituto de Salud Carlos III, Av. Monforte de Lemos, Madrid, Spain

**Keywords:** Cell death in the nervous system, Huntington's disease, Molecular neuroscience

## Abstract

Huntington’s disease (HD) is a progressive, autosomal dominant neurodegenerative disorder characterized by the selective dysfunction and loss of neurons in the striatum and cerebral cortex. Experimental evidence suggests that GABAergic medium-sized spiny neurons (MSNs) in the striatum are particularly vulnerable to glutamate-induced toxicity (excitotoxicity) and its analogues. However, the molecular mechanisms underlying MSN-specific death in HD remain poorly understood. The serine/threonine protein kinase D1 (PKD1) confers neuroprotection in various neuropathological conditions, including ischemic stroke. While excitotoxicity inactivates PKD1 in cortical glutamatergic neurons without altering its levels, active PKD1 potentiates the survival of excitatory neurons in highly excitotoxic environments. Here, we investigated whether PKD1 activity dysregulation contributes to MSN death in HD and its association with neurodegeneration. We found an unexpected reduction in PKD1 protein levels in striatal neurons from HD patients. Similarly, the R6/1 mouse model of HD exhibited progressive PKD1 protein loss, commencing at early disease stages, accompanied by decreased *Prkd1* transcript levels. PKD1 downregulation also occurred in the cerebral cortex of R6/1 mice, but only at late stages. Functionally, pharmacological PKD inhibition in primary striatal neurons exacerbated excitotoxic damage and apoptosis induced by glutamate N-methyl D-aspartate (NMDA) receptors, whereas expression of constitutively active PKD1 (PKD1-Ca) conferred neuroprotection. Furthermore, PKD1-Ca protected against polyQ-induced apoptosis in a cellular model of HD. In a translational approach, intrastriatal lentiviral delivery of PKD1-Ca in symptomatic R6/1 mice prevented the loss of DARPP-32, a molecular marker of MSNs. Collectively, our findings strongly suggest that PKD1 loss-of-function contributes to HD pathogenesis and the selective vulnerability of MSNs. These findings position PKD1 as a promising therapeutic target for mitigating MSN death in HD.

## Introduction

Huntington’s Disease (HD) is a fatal inherited neurodegenerative disorder characterized by motor, cognitive, and psychiatric symptoms [1]. HD is caused by a cytosine-adenine-guanine (CAG) repeat expansion within the coding region of the huntingtin gene (*HTT*), that generates an aberrant form of the protein containing an extended polyglutamine (polyQ) stretch [2]. Despite the ubiquitous expression of HTT, cell degeneration caused by mutant HTT (mHTT) primarily affects the brain. Early-stage pathology prominently features striatal damage, including the loss of inhibitory medium-sized spiny neurons (MSNs), and then spreads to the cerebral cortex as disease progresses [3, 4].

GABAergic MSNs are the most abundant cell type in the striatum constituting nearly 95% of all striatal neurons in rodents [5]. While multiple pathophysiological processes contribute to striatal neurodegeneration triggered by mHTT, the exact cause of the selective vulnerability of striatal MSNs in HD remains elusive [6]. Since MSNs receive numerous cortical and thalamic glutamatergic afferents [7], a long-standing hypothesis proposed an initial overactivity of glutamate neurotransmission triggering excitotoxicity in HD. Furthermore, several studies have shown functional alterations in specific glutamate receptors and decreased expression of glutamate transporters in both HD transgenic mouse models and HD patients. For example, diminished expression of the astrocytic glutamate uptake transporter, GLT1, has been observed in the striatum of the R6/1 mouse model of HD [8] and in human post-mortem tissue [9], potentially contributing to elevated glutamate levels. Consequently, overstimulation of the ionotropic glutamate receptor N-methyl D-aspartate (NMDAR) leads to neuronal death [10], particularly via activation of extrasynaptically located NMDARs [11]. In addition, this glutamate excitotoxicity is associated with dysregulation of intracellular Ca^2+^ homeostasis and activation of the calcium-dependent cysteine protease calpain, two connected events that synergistically cause neuronal death [12]. Indeed, several studies have demonstrated the protective role of calpain inhibition in HD mouse models [13, 14]. All these findings suggest a key role of excitotoxicity in HD pathology.

The serine/threonine protein kinase D1 (PKD1), encoded by the *PRKD1* gene [15], is a member of the calcium/calmodulin-dependent protein kinase superfamily, alongside PKD2 and PKD3 [16]. PKD1 activation, commonly assessed by Ser^916^ autophosphorylation [17], requires dual phosphorylation of Ser^744^ and Ser^748^ residues in its catalytic activation loop by various PKCs, depending on cell type and stimuli [18]. For example, in cultured dopaminergic neurons, oxidative stress, a common pathological process in neurodegenerative disorders, activates PKD1 via a well-characterized pathway involving caspase-3-mediated proteolytic activation of PKCδ, which in turn, activates PKD1 by phosphorylating the active loop residues. PKD1 activation also participates in neuronal polarity establishment, learning, and neuroprotection [19]. Our recent work demonstrated that excitotoxicity provokes early dephosphorylation-dependent inactivation of PKD1 in cortical neurons, shutting off an essential oxidative stress detoxification pathway, and ultimately compromising neuronal survival [20]. Conversely, expression of a constitutively active PKD1 (PKD1-Ca) mutant, resistant to dephosphorylation, confers neuroprotection against excitotoxicity and oxidative stress damage [20]. Disruption of the delicate equilibrium between protein phosphatases and kinases is among the multiple cellular dysfunctions associated with mHTT-mediated neuronal death [21]. Notably, PKCδ is early downregulated in HD [22], suggesting that PKD1 activity may also be altered in this neurodegenerative disease, compromising its hypothetical neuroprotective effect on inhibitory striatal MSNs.

Here, we have investigated PKD1 expression dynamics in HD striatum and cerebral cortex. Strikingly, we observed an unexpected decrease in PKD protein content in striatal neurons of HD patients. Brain samples from the R6/1 mouse model of HD exhibited a similar decline in PKD1 levels at both early and late symptomatic stages. Importantly, pharmacological inhibition of PKD in cultured mouse striatal MSNs enhanced their vulnerability to excitotoxic insults. We also provide evidence for the neuroprotective effects of PKD1-Ca against NMDA-induced excitotoxicity in primary cultured striatal neurons and against toxicity induced by mHTT in an HD cellular system. Finally, in vivo lentiviral transduction of PKD1-Ca in the striatum of aged R6/1 mice prevented the loss of DARPP-32, a key marker of MSNs.

## Results

### Decreased neuronal PKD levels in human HD brain

**Fig. 1:**
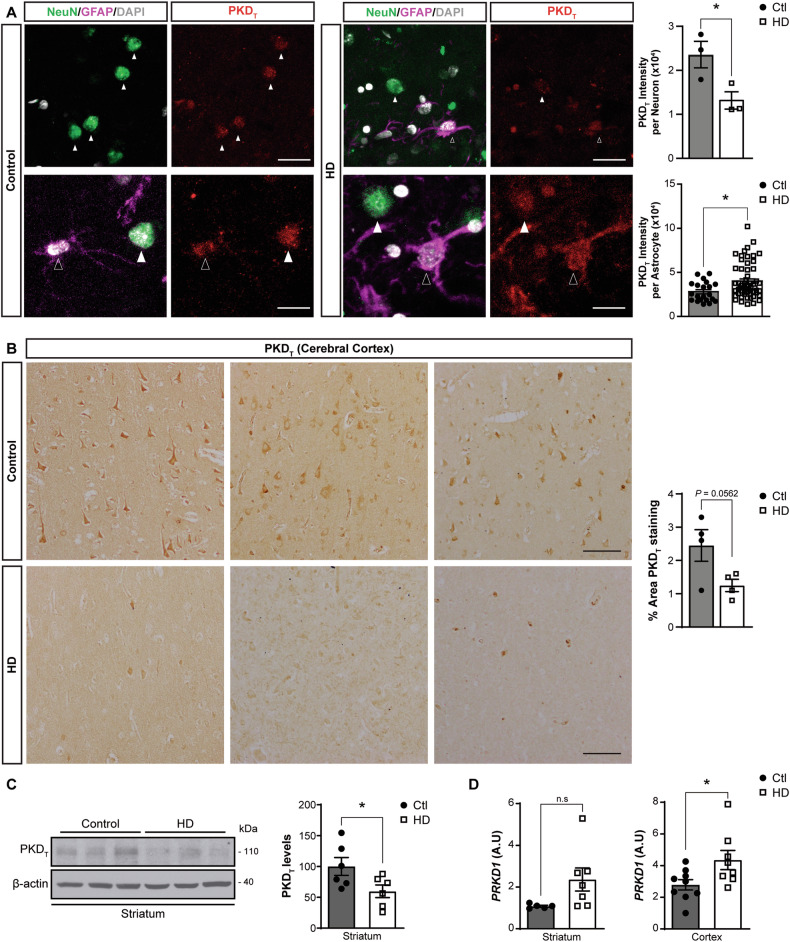
Decreased neuronal PKD levels in striatum from HD patients. **A** Representative confocal microscopy images of PKD_T_ staining (red channel) in neurons (NeuN^+^, green channel, full arrowhead) and astrocytes (GFAP^+^, purple channel, empty arrowhead) in striatum from HD patients and control individuals. Scale bar: 40 µm (top panels) and 20 µm (bottom panels). The top graph represents the quantification of PKD_T_ intensity staining in striatal neurons of control individuals compared to HD patients (*n* = 3 individuals per group). The bottom graph shows the quantification of PKD_T_ staining intensity in striatal astrocytes from control individuals compared to HD patients (21 and 58 GFAP-positive striatal astrocytes in control and HD samples, respectively). Nuclei were stained with DAPI. **B** PKD_T_ immunohistochemistry in cerebral cortex sections from HD patients and control individuals. Each panel is a representative image from different individuals Scale bar: 100 µm. Chromogen detection was quantified and expressed as the percentage of area stained by PKD_T_ from sections (*n* = 4 individuals per group). **C** Representative immunoblot of PKD_T_ and quantification of protein levels in homogenates from striatum necropsies from HD patients (*n* = 6) and control non-affected individuals (*n* = 6). Levels of β-actin were used as loading control for normalization purposes. **D** qRT-PCR analysis of *PRKD1* mRNA from striatum and cortex of HD patients (*n* = 6–8) and non-affected individuals (*n* = 5–9). Data are represented as mean ± SEM. *P < 0.05 or n.s. (not significant) using unpaired Student’s *t* test.

We have demonstrated recently that excitotoxic insults trigger the shut-off of a constitutive pro-survival oxidative stress detoxification pathway in neurons mediated by PKCδ-PKD1 [20]. Here, we hypothesized that pathological events associated with HD, such as excitotoxicity [6] or the early down-regulation of PKCδ [22], may lead to PKD1 inactivation, consequently losing its neuroprotective ability and thereby promoting neuronal death. To analyse the distribution of PKD in human striatum and its possible HD-associated neuropathology, we triple stained striatal sections from HD patients and control individuals with an antibody against PKD together with antibodies recognizing specific markers for neurons (NeuN) and astrocytes (GFAP). Immunofluorescence and confocal microscopy analysis showed that neurons in the striatum from control subjects contained PKD (Fig. 1A, top panels and graph). In contrast, the striatum from HD patients showed PKD presence predominantly in GFAP^+^ reactive astrocytes (Fig. 1A, bottom panels and graph), accompanied by a substantial reduction in neuronal PKD levels, as evidenced by quantification of the kinase immunofluorescence signal in GFAP^+^ and NeuN^+^ cells, respectively (Fig. 1A). The PKD immunohistochemical signal in cortical paraffin sections from control individuals was predominantly neuronal, whereas in the HD cortex staining tended to be lower and appeared enriched in a cell population with a morphology distinct from that of neurons (Fig. 1B).

To validate these findings, we performed immunoblot analysis of protein extracts, revealing a marked decrease in PKD levels in the striatum of HD patients compared to control subjects (Fig. 1C). Unfortunately, we failed to detect PKD autophosphorylated at serine 916 (p-S916) as a readout of active kinase [17] in these protein extracts, probably due to the post-mortem nature of human samples and the sensitivity of phosphorylated residues to the action of post-mortem activated phosphatases [23].

Next, we investigated whether transcriptional inhibition could account for the decreases in PKD protein levels. Contrary to expectations, analysis by qRT-PCR of *PRKD1* mRNA content in the striatum and cortex of HD patients and control subjects showed a trend towards an increase in HD striatum, with a significant upregulation of *PRKD1* transcripts observed in HD cortex (Fig. 1D). Given that the PKD antibody can detect the highly homologous C-terminal end of PKD1 and PKD2, we also quantified *PRKD2* mRNA in both brain structures, finding no significant changes (Supplementary Fig. 1A). Together, these results suggest that the decrease in PKD protein in striatum of HD patients depends on post-transcriptional mechanisms.

### Striatal decrease of PKD in the R6/1 mouse model of HD

**Fig. 2:**
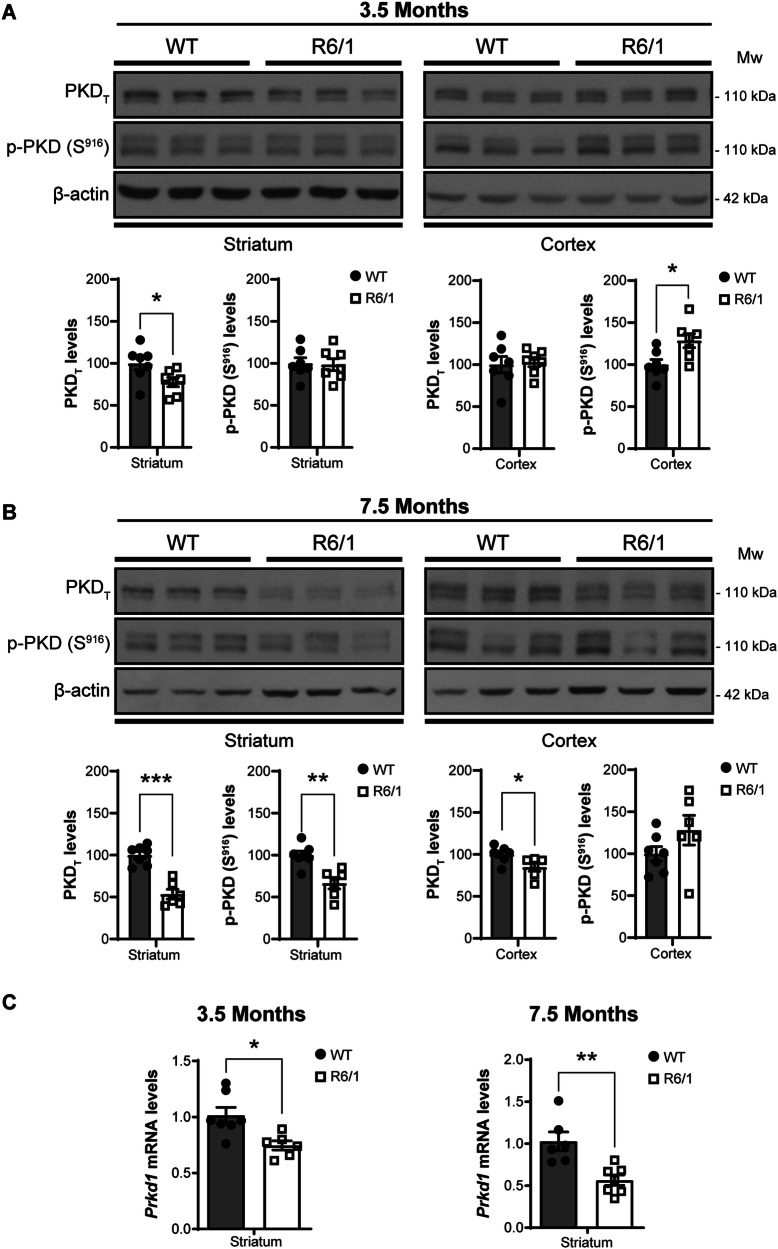
Early loss of striatal PKD in R6/1 mouse model of HD. Representative immunoblot of PKD_T_ and p-PKD (S^916^) and quantification of protein levels in homogenates from striatum and cerebral cortex of 3.5- (**A**) or 7.5-month-old (**B**) R6/1 (*n* = 6) and wild type (WT) mice (*n* = 7) of the same ages. Levels of β-actin were used as loading control for normalization purposes. **C** qRT-PCR analysis of *PRKD1* mRNA from striatum of 3.5-month-old or 7.5-month-old R6/1 (*n* = 6–7) and WT (*n* = 6–7) mice. Data are represented as mean ± SEM. *P < 0.05, **P < 0.01 or ***P < 0.001 using unpaired Student’s *t* test.

We investigated whether the R6/1 mouse model of HD displayed similar changes in PKD as those observed in the brains of HD patients. To assess brain protein expression during disease progression, we used 3.5- and 7.5-month-old R6/1 mice, corresponding to early symptomatic and late stages of the disease, respectively [24]. Immunoblot analysis of protein levels showed significant (*P* < 0.05) decreases in PKD signal in the striatum at early stages (Fig. 2A), registering more pronounced differences as the disease progressed (Fig. 2B). The cerebral cortex only registered significant PKD decreases (*P* < 0.05) at late disease stages (Fig. 2B). Notably, PKD levels remained stable across both age groups in wild type (WT) mice in both brain structures (Supplementary Fig. 2). Unlike human necropsy samples, autophosphorylated active PKD was detectable by immunoblotting with the p-S916 antibody in mouse brain samples. PKD activity, determined as p-S916 levels, also decreased in the R6/1 striatum at late disease stages, coinciding with major total PKD protein downregulation (Fig. 2A, B). Surprisingly, p-S916 signal increased in protein extracts from R6/1 mouse cerebral cortex at early symptomatic stages, suggesting an initial transient rise in PKD activity in this brain structure. In contrast to what was observed in striatal samples from HD patients, the decrease of PKD protein in the striatum of R6/1 mice could be attributed to a transcriptional alteration, as qRT-PCR analysis revealed significantly lower *Prkd1* mRNA content in this brain region at both disease stages studied (Fig. 2C). More precisely, *Prkd1* transcripts in R6/1 mouse striatum showed a significant decrease of nearly 25% (*P* < 0.05) at early stages, becoming more pronounced at late stages, reaching a 45% (*P* < 0.01) reduction (Fig. 2C). Since *Prkd2* mRNA striatal levels increased in 7.5-month-old R6/1 mice (Supplementary Fig. 1B), these data strongly suggest that the downregulation of PKD protein levels in the R6/1 mouse striatum may primarily result from *Prkd1* transcriptional inhibition.

### Neuronal PKD loss in the striatum of early symptomatic R6/1 mice

**Fig. 3:**
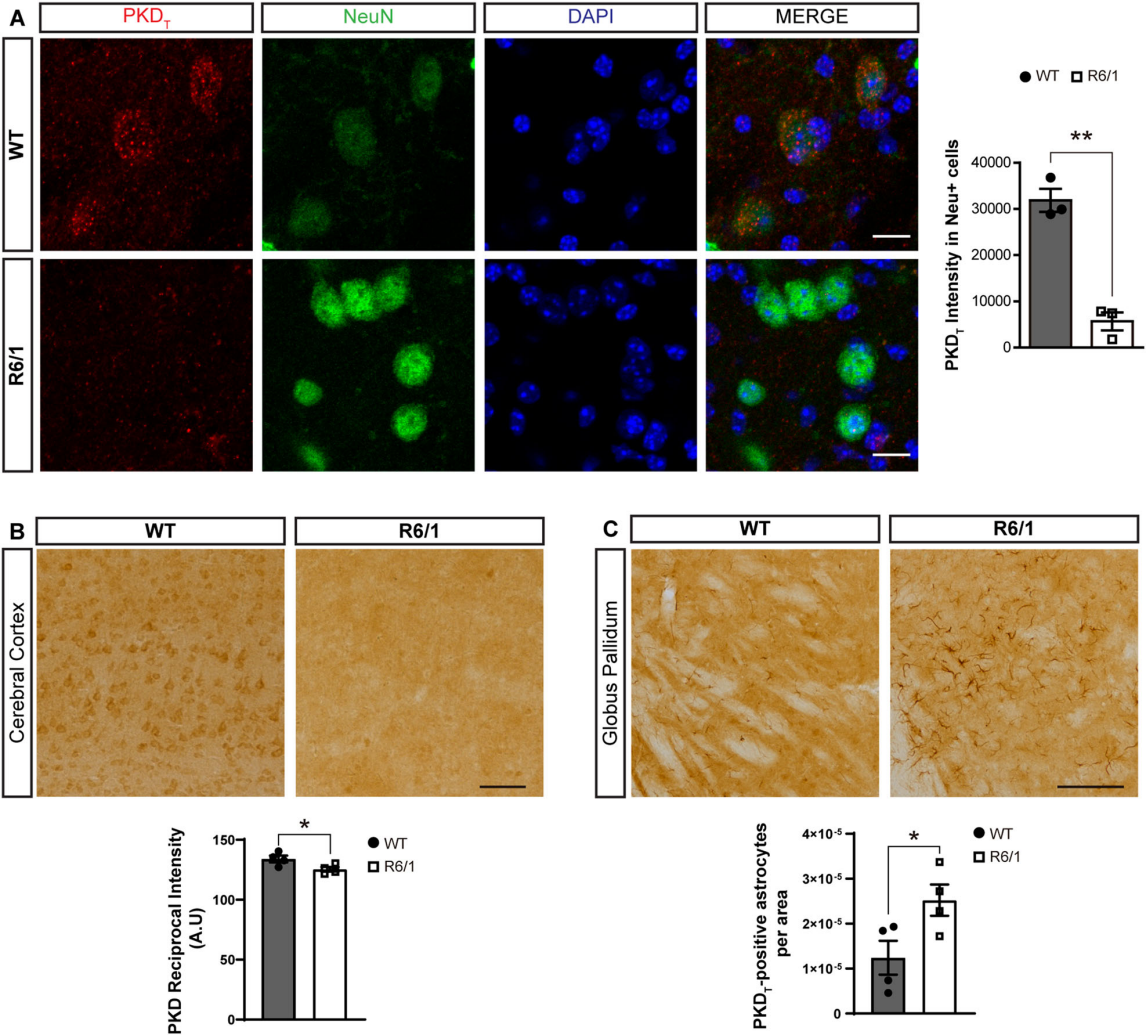
Early loss of striatal neuronal PKD in R6/1 mice occurs mainly in neurons while PKD expression appears in astrocytes. **A** Representative confocal microscopy images of PKD_T_ (red channel) in neurons (NeuN^+^, green channel) in striatum of 3.5-month-old WT and R6/1 mice. Nuclei were stained with DAPI. Scale bar: 20 µm. Graph represents the quantification of PKD_T_ intensity staining in striatal neurons of WT compared to R6/1 mice (3 animals per group). PKD_T_ immunohistochemistry in cerebral cortex (**B**) and globus pallidum (**C**) sections from 7.5-month-old WT and R6/1 mice. Scale bar: 150 µm. Graphs represent the quantification of chromogen detection and was expressed as DAB-staining reciprocal intensity (Arbitrary Units, A. U.) (**B**) or quantification of PKD_T_-positive astrocytes per area (**C**) (4 animals per group). Data are represented as mean ± SEM. * *P* < 0.05 or ** *P* < 0.01 using unpaired Student’s *t* test.

To identify the cell type where PKD expression was lower in the initial stages of the disease, we performed immunofluorescence staining of PKD together with markers for neurons (NeuN) and astrocytes (GFAP) on brain sections from 3.5-month-old R6/1 mice. Low magnification confocal microscopy images revealed an overall decrease in PKD fluorescent signal in R6/1 striatum compared to WT (Supplementary Fig. 3A) and quantitative analysis of higher magnification images confirmed that this robust decline at early symptomatic stages takes place in neurons (Fig. 3A). Such decrease could also be hinted by immunohistochemistry for the active kinase with the p-S916 antibody (Supplementary Fig. 3B) despite yielding a quite faint signal.

The loss of PKD staining was also evident by immunohistochemistry in the cortex of 7.5-month-old R6/1 mice compared to WT animals (Fig. 3B). Strikingly, at this age, we observed an increase in the number of PKD-stained cells with astrocytic-like morphology in the globus pallidum of R6/1 compared to WT mice (Fig. 3C). However, this type of labeling is absent in the striatum where only subtle PKD staining in cells with neuron-like morphology is found (Supplementary Fig. 3C). This observation suggests a complex regulation of PKD expression in HD pathology, diminishing in certain brain regions and cell types while increasing in others. Together, our data indicate that loss of PKD expression takes place in striatal neurons of this HD mouse model at early stages, suggesting that this step could contribute to initiate neuronal cell death programs.

### PKD inhibition enhances excitotoxicity in cultured rat primary GABAergic striatal neurons

**Fig. 4:**
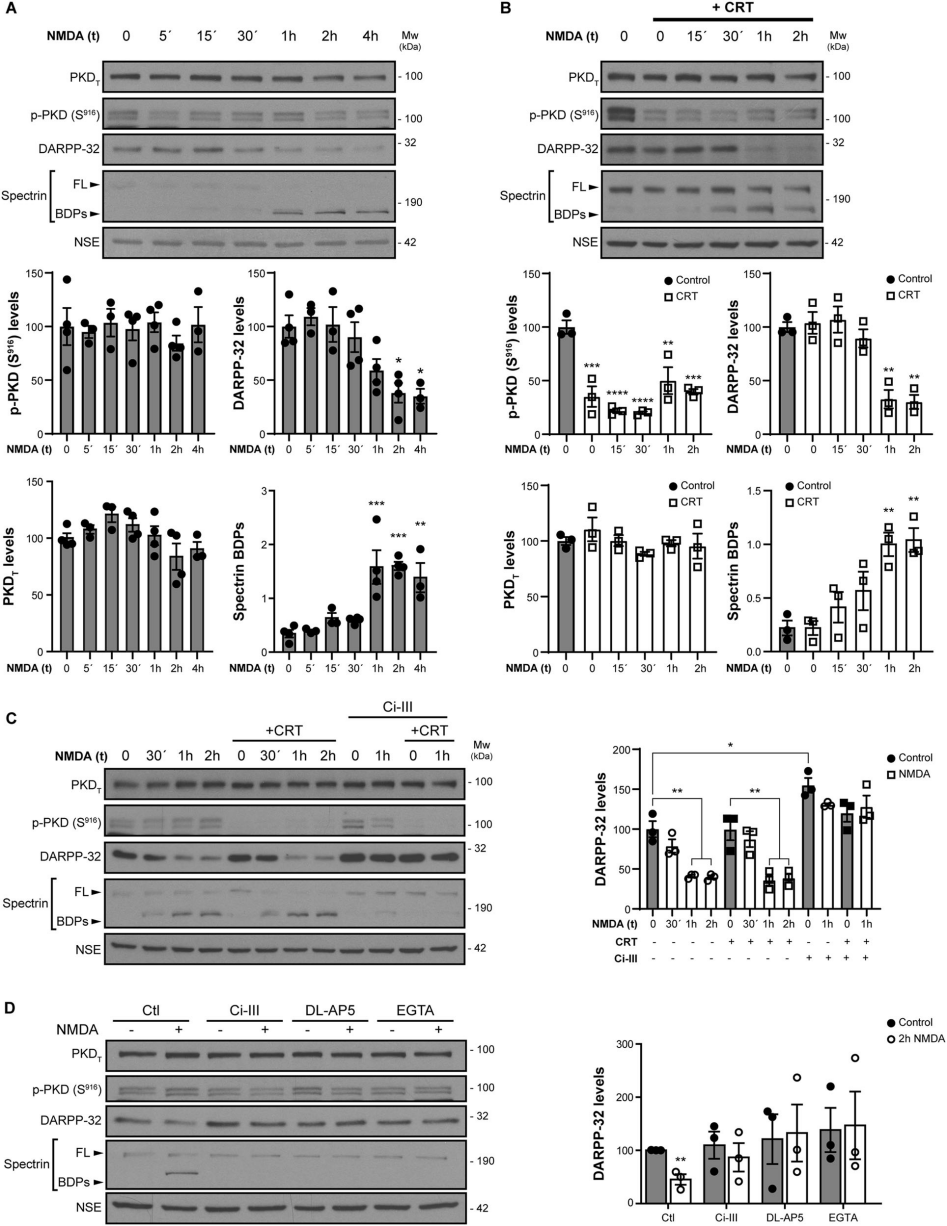
PKD inhibition enhances excitotoxicity-induced DARPP-32 loss in cultured rat primary striatal neurons by mechanisms dependent on NMDARs, calcium and calpain. PKD_T_, p-PKD (S^916^) and DARPP-32 immunoblot analysis of cultured primary mature striatal neurons stimulated with excitotoxic concentrations of NMDA (50 µM) plus its co-agonist glycine (10 µM) (a treatment referred hereafter as “NMDA”) for the indicated times (**A**–**C**), in combination with the PKD-specific pharmacological inhibitor CRT0066101 (CRT, 5 µM) 1 h before NMDA-treatment (**B**, **C**) or together with the calpain inhibitor Ci-III (20 µM) (**C**) and remained in the culture media for the duration of the experiment. Spectrin full-length (FL) and break-down products (BDPs) confirming excitotoxicity and calpain activation are also shown. Graphs represent the quantification of PKD_T_, p-PKD (S^916^), spectrin BDPs (**A**, **B**) and DARPP-32 (**A**–**C**) immunoblot signal relative to loading control neural-specific enolase (NSE). *n* = 3–4 independent experiments. **D** PKD_T_, p-PKD (S^916^) and DARPP-32 immunoblot analysis of cultured striatal neurons pre-treated for 1 h with the calpain inhibitor Ci-III, the NMDAR antagonist DL-AP5 or the Ca^2+^ chelator EGTA, and then stimulated with NMDA for 2 h. Graph represents the quantification of DARPP-32 immunoblot signal relative to loading control NSE. *n* = 3 independent experiments. All data are represented as mean ± SEM. *P < 0.05, **P < 0.01, ***P < 0.001 or ****P < 0.0001 using one-way ANOVA, followed by Bonferroni’s post hoc test.

Recent studies from our group demonstrated that PKD inactivation causes the death of excitatory glutamatergic neurons and potentiates NMDARs-mediated excitotoxicity [20]. Since NMDARs and excitotoxicity also play a key role in striatal GABAergic inhibitory MSNs death in HD [6], we investigated whether PKD inhibition could enhance excitotoxic death in cultured mature rat primary striatal neurons. For these studies, we selected a 5 μM dose of the PKD-specific pharmacological inhibitor CRT0066101 (CRT), confirming that it efficiently inhibited PKD activity without altering total PKD content or disrupting the molecular identity of MSNs, as indicated by stable expression of the MSN-specific marker DARPP*-*32 (Fig. 4) [25].

To induce excitotoxicity, the striatal neuronal cultures were stimulated with excitotoxic concentrations of the NMDARs agonist NMDA (50 μM) and its co-agonist glycine (10 μM), a treatment referred to here as “NMDA”, for varying time periods. Unlike our previous observations in cultured glutamatergic cortical neurons [20], immunoblot analysis of lysates from cultured striatal neurons showed no substantial alterations in total PKD levels or its activity, as assessed by p-S916 signal, across all NMDA-time points analysed (Fig. 4A). However, NMDA treatment for 2 h and 4 h significantly reduced DARPP*-*32 levels to approximately 40% (*P* < 0.05) relative to untreated cells (Fig. 4A). Treatment of striatal neurons with CRT alone (5 μM) decreased basal PKD activity, as expected, without altering total PKD content or that of DARPP*-*32 (Fig. 4B). Importantly, the combination of CRT with NMDA accelerated and enhanced DARPP-32 downregulation, with levels decreasing to ~33% (*P* < 0.01) relative to control cultures just 1 h after NMDA addition (Fig. 4B; compare DARPP-32 quantification graph with that in Fig. 4A). Excitotoxicity was confirmed by the cleavage of full-length (FL) brain spectrin to breakdown products (BDPs) by calpain, a Ca^2+^-dependent protease activated by NMDARs overstimulation [26] (Fig. 4). We then investigated the mechanism involved in DARPP-32 decay. Pretreatment of striatal neurons with the calpain inhibitor Ci-III blocked the excitotoxicity-induced loss of this marker, both in the presence and absence of CRT (Fig. 4C). In addition, pretreatment with the NMDAR antagonist DL-AP5 or the calcium chelator EGTA also preserved DARPP-32 levels (Fig. 4D). However, while Ci-III, DL-AP5, and EGTA prevented spectrin processing, they did not alter PKD levels or activity (Fig. 4D and Supplementary Fig. 4). Collectively, these data indicate that DARPP-32 is degraded in cultured rat MSNs under excitotoxic conditions via NMDARs-, Ca^2+^- and calpain-dependent mechanisms, and that PKD inhibition accelerates and exacerbates this process.

**Fig. 5:**
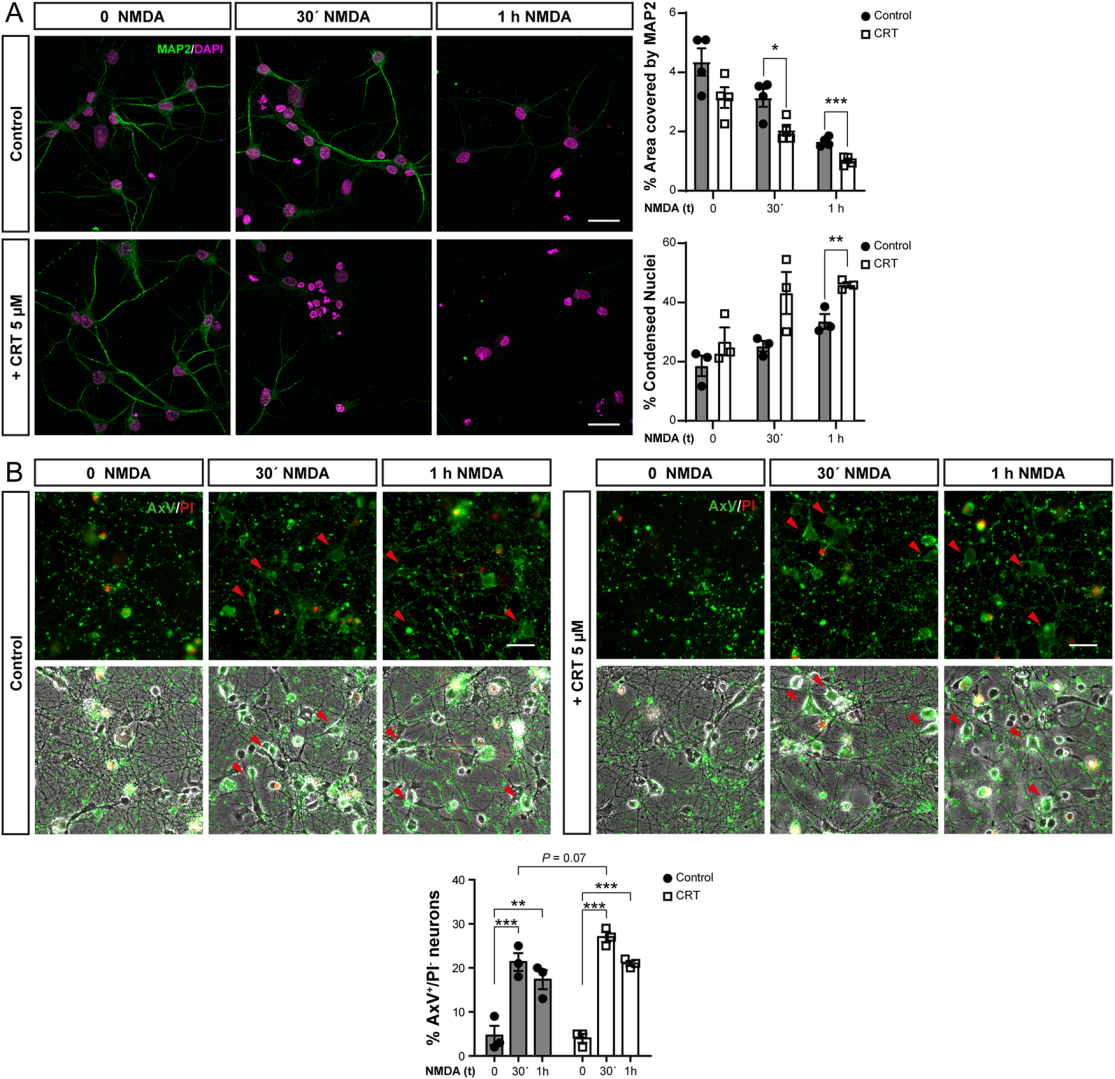
PKD inhibition enhances excitotoxic cell death of cultured striatal neurons. **A** Representative confocal microscopy images of cultured primary mature striatal neurons (MAP2^+^ cells, green channel) stimulated with NMDA in combination with CRT for the indicated times. Nuclei were stained with DAPI. Scale bar: 40 µm. Graphs represents the percentage of area covered by MAP2 staining and the percentage of condensed nuclei (*n* = 3 independent experiments). **B** Representative fluorescence microscopy (top panels) and corresponding brightfield images (bottom panels) of cultured primary mature striatal neurons stimulated with NMDA in combination with CRT for the indicated times. After treatment, neurons were stained with Annexin V-FITC (AxV) and Propidium Iodide (PI). Scale bar: 20 µm. The graph represents the percentage of early apoptotic neurons (AxV^+^/PI^-^) relative to the total number of neurons in each condition (*n* = 3 independent experiments). All data are represented as mean ± SEM. *P < 0.05, **P < 0.01 or ***P < 0.001 using one-way ANOVA, followed by Bonferroni’s post hoc test.

Next, to examine the effect of PKD inhibition on neuronal death, we treated striatal neuronal cultures with NMDA alone or in combination with CRT for 30 min or 1 h and quantified the area covered by the neuronal specific marker MAP2 as an indicator of neuronal integrity. CRT alone did not substantially alter MAP2 staining but potentiated NMDA-induced neuronal loss (Fig. 5A). We also quantified condensed nuclei as an indication of neuronal damage. Again here, CRT treatment showed no marked effects, but in combination with NMDA, it increased the number of cells presenting nuclear condensation at the longer time point studied (Fig. 5A). These results indicate that while PKD inactivation does not affect the basal survival of cultured striatal neurons within the studied time frame, it increases their vulnerability to excitotoxicity.

To further characterize the cell death processes associated with excitotoxicity [27] that may compromise striatal neuron survival in response to CRT and NMDA, we assessed early apoptosis using annexin V (AxV) binding assays in adherent cells analysed by fluorescence microscopy. Striatal neurons grown on coverslips were pre-treated with CRT for 1 h or left untreated, followed by NMDA stimulation for 30 min or 1 h. Preincubation with CRT alone did not increase AxV labeling (Fig. 5B). In contrast, NMDA treatment induced a highly significant increase in AxV binding, with the most pronounced and statistically significant effects observed at the earliest time point (30 min) following NMDA stimulation (Fig. 5B). Notably, while AxV binding tended to be higher in neurons pretreated with CRT compared to those treated with NMDA alone, this difference did not reach statistical significance (*P* = 0.07) (Fig. 5B).

To further investigate the potential role of CRT in neuronal apoptosis, we assessed the levels of active caspase-3 (AC3) and the cleaved fragment of its substrate PARP-1, both well-established markers of apoptotic activation. Immunoblot analysis of protein lysates demonstrated a substantial increase in both molecules when cultured striatal neurons were treated with CRT up to 48 h (Supplementary Fig. 5). Together, these findings provide evidence that CRT, and therefore PKD inhibition, contributes to the apoptotic death of striatal neurons.

### Neuroprotection of striatal neurons against NMDA-induced excitotoxicity by PKD1

**Fig. 6:**
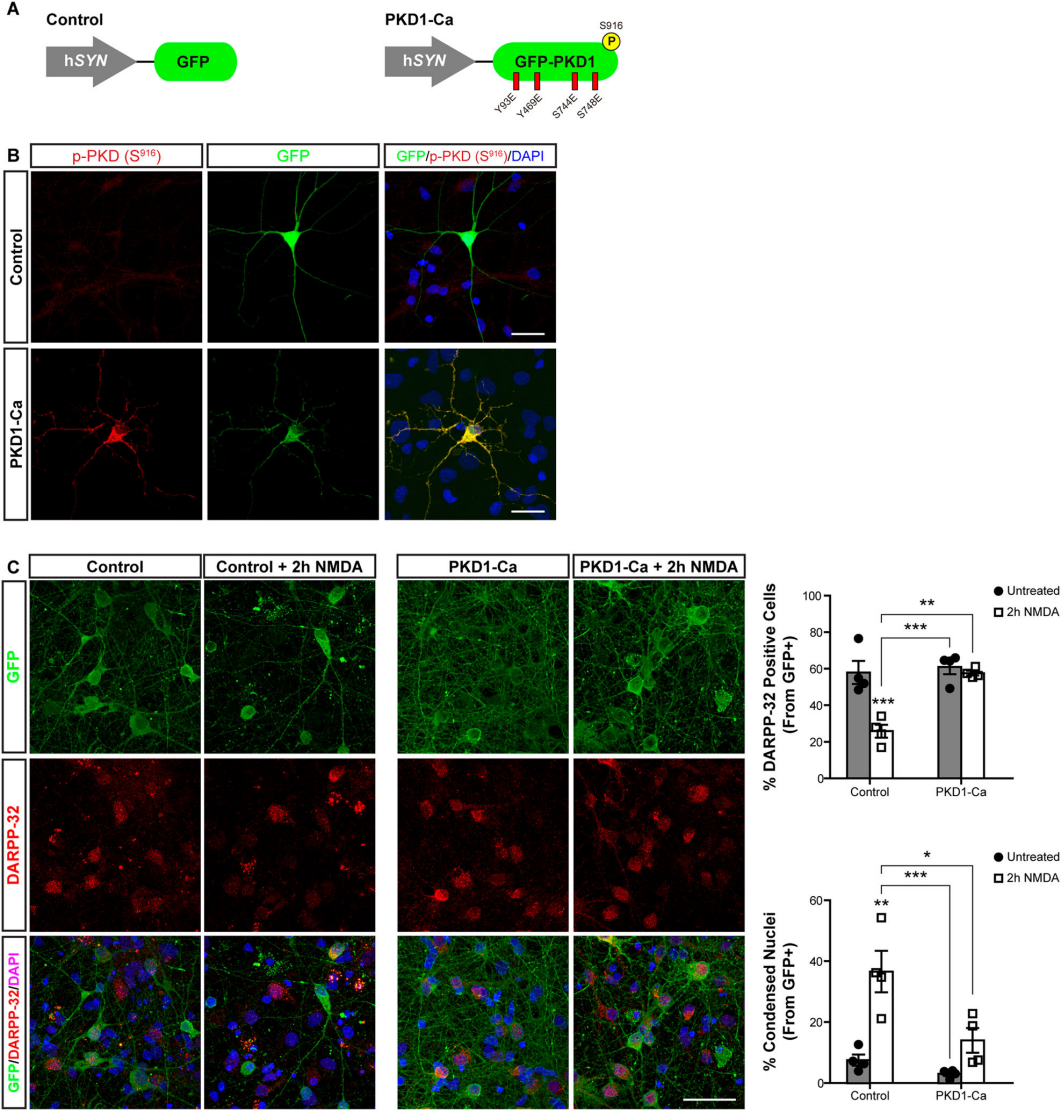
Neuroprotection of cultured striatal neurons against NMDA-induced excitotoxicity by PKD1. **A** Scheme of the vector construct used for neuronal expression of a constitutively active PKD1 mutant (PKD1-Ca). GFP alone or fused to PKD1-Ca was cloned under the neurospecific human synapsin promoter (h*SYN*). **B** Representative confocal microscopy images of cultured rat primary mature striatal neuron transfected with GFP (Control) or PKD1-Ca and stained with p-PKD (S^916^) and DAPI. Scale bar: 25 µm. **C** Representative confocal microscopy images of GFP, DARPP-32 and DAPI signal of neurons transduced with lentiviral particles for GFP and GFP-PKD1-Ca expression treated or not with NMDA for 2 h. Scale bar: 40 µm. Top Graph represents the percentage of DARPP-32^+^ surviving neurons after NMDA treatment, relative to total GFP^+^ neurons in untreated conditions. Bottom graph represents the percentage of GFP^+^ neurons bearing condensed nuclei relative to total GFP^+^ neurons (*n* = 20–60 neurons per condition; *n* = 4 independent experiments). All data are represented as mean ± SEM. *P < 0.05, **P < 0.01 or ***P < 0.001 using one-way ANOVA, followed by Bonferroni’s post hoc test.

The decreased neuronal levels of PKD in the striatum of HD patients and R6/1 mice, coupled with the deleterious effects of PKD inhibition in cultured striatal neurons under excitotoxic conditions, suggested that increasing PKD activity might confer neuroprotection to these neurons. To test this hypothesis, we investigated whether the expression of a constitutively active PKD1 mutant (PKD1-Ca) could protect striatal neurons from excitotoxic death. We previously generated the PKD1-Ca mutant by substituting four critical residues involved in PKD activation (Tyr93, Tyr469, Ser744, and Ser748) by glutamic acid, mimicking their phosphorylated state and rendering the kinase constitutively active [20]. PKD1-Ca was expressed fused to GFP under the control of the human synapsin neurospecific promoter (h*SYN*), while GFP alone under the same promoter served as a control (Fig. 6A). First, we confirmed that striatal neurons transfected with PKD1-Ca plasmid exhibited high levels of active PKD1 compared to those transfected with the GFP control plasmid, as indicated by p-S916 immunofluorescence (Fig. 6B). Confocal microscopy-based quantification of DARPP-32 staining and GFP fluorescence revealed that PKD1-Ca expression protected striatal neurons from NMDA-induced excitotoxicity (Supplementary Fig. 6). While only 20% of GFP-expressing control neurons retained DARPP-32 staining after 2 h of NMDA treatment, 50% of PKD1-Ca-expressing neurons remained DARPP-32^+^, suggesting enhanced survival under excitotoxic conditions (Supplementary Fig. 6). The neuroprotective role of PKD1 was further validated in cultured striatal neurons transduced with lentiviral particles encoding GFP or GFP-PKD1-Ca (Supplementary Fig. 7). In these transduced neurons, untreated or treated with NMDA for 2 h, we quantified GFP^+^ cells (expressing GFP or GFP-PKD1-Ca) that were alive and DARPP-32^+^. As shown in Fig. 6C, the number of DARPP-32^+^ neurons remained similar in control, untreated cultures expressing either GFP or GFP-PKD1-Ca. However, NMDA treatment markedly decreased the number of DARPP-32^+^ cells in GFP-expressing neurons, whereas those transduced with GFP-PKD1-Ca were largely protected (Fig. 6C). Additionally, analysis of nuclear condensation in GFP^+^ cells revealed a substantial increase in apoptotic neurons in the GFP control group following NMDA treatment (Fig. 6C). Together, these findings strongly suggest that PKD1 exerts a potent neuroprotective action in striatal neurons, safeguarding them from neurotoxic stimuli (or excitotoxicity) in vitro.

### PKD1 protects from polyQ-induced apoptosis in an HD cell model

**Fig. 7:**
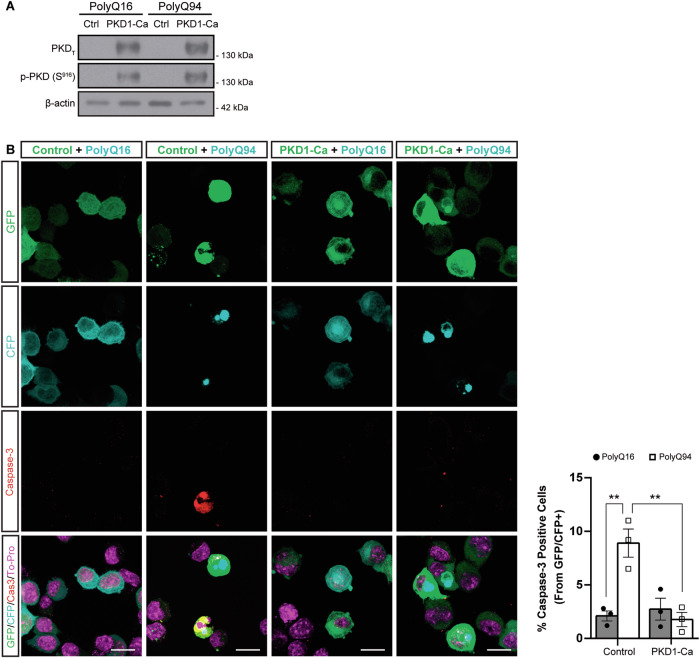
PKD1 protects from polyglutamine-induced apoptosis in a cell model of HD. **A** Representative immunoblot of PKD_T_ and p-PKD (S^916^) in homogenates from neuroblastoma N2a cell line transfected with PolyQ16 or PolyQ94 in combination with p-EF-BOS-GFP (Ctrl) or p-EF-BOS-PKD1-Ca (PKD1-Ca). Levels of β-actin were used as loading control. **B** Representative confocal microscopy images of GFP, CFP, and cleaved caspase-3 in the neuroblastoma N2a cell line transfected as described in (**A**). Nuclei were stained with To-Pro. Scale bar: 10 µm. Graph represents the quantification of cleaved caspase-3^+^ cells relative to GFP/CFP^+^ cells 24 h post-transfection (*n* = 25 cells per condition; *n* = 3 per group). All data are represented as mean ± SEM. **P < 0.01, using one-way ANOVA, followed by Bonferroni’s post-hoc test.

Once demonstrated that PKD1-Ca protected striatal neurons against excitotoxic insults, we investigated whether it could also mitigate the mHTT polyQ-induced apoptosis characteristic of HD. For these studies, we selected the neuroblastoma N2a cell line as a model system since they have been reported to undergo apoptosis upon transfection with exon1 mHTT plasmids [28]. We transfected exon 1 of human *HTT* containing 16 or 94 polyQ extensions (PolyQ16 or PolyQ94, respectively), both fused to CFP, in N2a cells and examined the neuroprotective effect of co-transfecting PKD1-Ca, by assessing cleaved caspase-3 as an apoptotic marker. First, we confirmed the elevated expression and corresponding high kinase activity of PKD1-Ca in N2a transfected cells using immunoblot analysis (Fig. 7A). Confocal microscopy analysis revealed that the expression of PolyQ94, as evidence by CFP signal, led to the appearance of cleaved caspase-3 immunofluorescence, evidencing as a read out of apoptosis induction (Fig. 7B). Notably, the incidence of apoptosis in cells co-transfected with PolyQ94 and PKD1-Ca was sharply lower and comparable to that of N2a cells transfected with non-toxic PolyQ16 (Fig. 7B). These data indicate that PKD1-Ca protects against mHTT-induced apoptosis in this HD cell model.

### PKD1 protects against HD-induced DARPP-32 decline in R6/1 mice

**Fig. 8:**
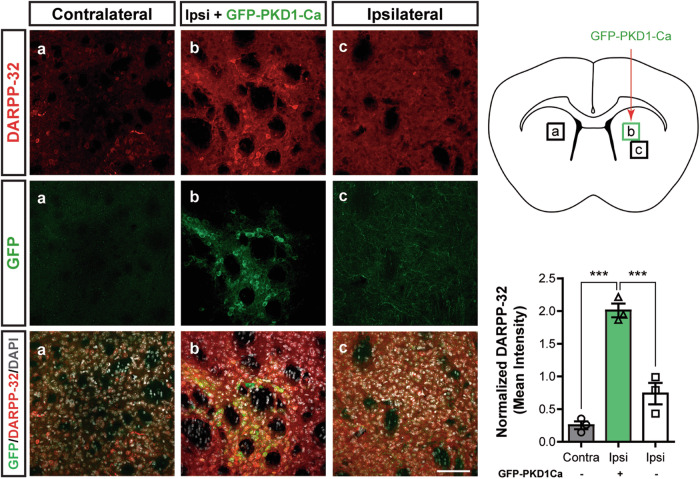
In vivo expression of GFP-PKD1-Ca enhances DARPP-32 levels in R6/1 striatum. Panels show representative confocal microscopy images of GFP, DARPP-32 and DAPI signal of contralateral and ipsilateral R6/1 striatum of 6-month-old mice injected with GFP-PKD1-Ca lentiviral particles. Graph represents normalized DARPP-32 mean intensity of GFP-PKD1-Ca transduced cells, the equivalent contralateral area and GFP-PKD1-Ca non-expressing neurons adjacent to transduced cells. All data are represented as mean ± SEM. ***P < 0.001, using one-way ANOVA, followed by Bonferroni’s post-hoc test.

To further investigate the neuroprotective role of PKD1 in vivo, we stereotaxically injected lentiviral particles encoding PKD1-Ca for neurospecific expression into one striatum of 6-month-old R6/1 mice (Fig. 8). In this HD mouse model, while MSNs are not overtly lost, DARPP-32 levels progressively decline as the disease progresses, making this reduction a hallmark of striatal degeneration in both HD patients and mice [29, 30]. Five weeks post-injection, we analysed striatal DARPP-32 expression by immunofluorescence. Our study revealed a substantial increase in DARPP-32 levels in areas enriched with PKD1-Ca transduced neurons (GFP^+^) compared to neighboring ipsilateral or contralateral regions lacking GFP^+^ cells (2.01 ± 0.11 PKD1-Ca vs. 0.74 ± 0.16 ipsilateral or 0.25 ± 0.06 contralateral) (Fig. 8). Notably, we also observed a trend toward increased DARPP-32 expression in regions adjacent to the transduction sites, suggesting a potential paracrine neuroprotective effect. Altogether, these in vivo results underscore a critical role for PKD1 in protecting against MSN damage and neurodegeneration in R6/1 mice.

## Discussion

Neurodegenerative disorders are characterized by death of selective neuronal subpopulations in specific brain regions. Identifying the precise molecular mechanisms mediating this selective vulnerability is essential for the development of novel neuroprotective therapies.

This is the first report identifying alterations in PKD1 expression in HD and investigating its potential role as a neuroprotective kinase in this condition. We have previously demonstrated that PKD1 is crucial for the survival of glutamatergic cortical neurons by promoting oxidative stress detoxification through the maintenance of mitochondrial superoxide dismutase 2 (SOD2) expression [20]. Our findings indicated that PKD1 activity, and consequently this neuroprotective pathway, is suppressed by excitotoxicity in both in vitro models and in vivo models of ischemic brain damage, as well as in human ischemic stroke patients [20]. Mechanistically, the excitotoxic inactivation of PKD1 involved the activation of phosphatases, while the levels of the kinase remained intact.

In contrast to these findings, we observed a substantial reduction in PKD protein levels in the striatum, the primarily affected brain structure in HD, with a clear trend toward decreased levels in the cerebral cortex of HD patients. Similarly, in brains from R6/1 mice, there was a selective downregulation of PKD protein in the striatum that initiated in early disease stages and intensified as the disease progressed, whereas decrease of PKD in the cortex was only evident at late-stage disease.

In the HD mouse model brain, decreases in PKD protein levels closely correlate with a decline in striatal *Prkd1* mRNA, while *Prkd2* expression remains unchanged or even increases in late stages, suggesting that PKD1 is the main affected isoform. Decrease of *Prkd1* transcripts in R6/1 mice begins in early stages, prior to observable neuronal death, indicating that PKD1 protein downregulation might result from transcriptional alterations, although a post-transcriptional contribution cannot be excluded. The regulatory mechanisms governing *Prkd1* gene expression are poorly understood. While epigenetic inactivation of *Prkd1* by hypermethylation of the gene promoter has been described in breast and gastric cancer [31**–**33], repression of *Prkd1* expression by nuclear β-catenin has been observed in colon and prostate cancer [34, 35]. To our knowledge, only one study has examined the transcriptional regulation of *Prkd1* in neurons. Using a dopaminergic neuronal system, this study demonstrated that NF-kB p65, Sp1, and Sp3 can transactivate the mouse *Prkd1* promoter and that epigenetic mechanisms, such as DNA methylation and histone acetylation, also regulate PKD1 gene expression [36]. In this line, HD is associated with extensive changes in DNA methylation in brain tissues [37, 38]; Sp1 and Sp3 transcriptional activity is also altered in the pathology [39, 40]; and mHTT downregulates NF-kB p65, inducing oxidative stress and cell death [41]. Importantly, we have previously reported that PKD1 inactivation in neurons leads to NF-kB p65 nuclear exclusion, downregulating its target genes, including the antioxidant enzyme gene *Sod2* [20]. Thus, the inhibition of the NF-kB pathway and PKD deficiency in HD may be interconnected in a feed-back loop, contributing to oxidative stress damage and neuronal loss induced by mHTT. Further studies are necessary to elucidate whether these mechanisms or others yet to be discovered account for the decreased *Prkd1* mRNA levels observed in R6/1 brains.

In human HD brain, striatal decline in PKD protein levels is not accompanied by *PRKD1* or *PRKD2* mRNAs downregulation. Instead, both transcripts exhibit a trend to increase, with significance reached only for *PRKD1* in the cerebral cortex. Of note, human HD samples are obtained at the terminal stage of the disease, where compensatory mechanisms, not yet evident in 7.5-month-old R6/1 mice, may have taken place. In addition, the differential behavior of non-neuronal cells such as reactive astrocytes could also contribute to interspecies differences, as astrogliosis is a hallmark of HD in human brain [42] while, as previously described, this is barely detected in the R6/1 mouse model [43]. Here, we have identified the significant presence of PKD in a discrete population of astrocytes located in the globus pallidum of R6/1 mice and in GFAP^+^ cells in the striatum of human HD brains. This finding represents the first report demonstrating the presence of PKD in human brain tissue not only within neurons but also in reactive astrocytes. Therefore, reactive astrocytes may, at least in part, account for the apparent discrepancies observed between human and mouse samples. Investigating the role of PKD in astrocyte biology under both physiological and pathological conditions will undoubtedly be an interesting area of future research.

Neurons are the cell type where the expression of PKD1 is predominantly detected in the brain and here we demonstrate that, in HD, PKD1 is in fact clearly downregulated in neurons. This decrease, observed in striatum from early symptomatic stages in R6/1 mice, suggests a causative role of PKD1 decrease in the etiology of the disorder. Striatal MSNs are the primarily affected neuronal population in HD. Their low levels of reactive oxygen species detoxifying systems, particularly SOD2, and increased expression of GluN2B subunits of NMDARs may indicate their higher vulnerability to oxidative stress and glutamate excitotoxicity in HD [44]. In our histological analysis of brain tissue from control individuals, we noticed that detection of PKD or its active form is much slower in striatal than in cortical tissue. It is therefore possible that MSNs contain very low levels of this kinase compared to cortical neurons, which may keep their *Sod2* expression repressed and contribute to their hypervulnerability in HD. In addition, neurons in the HD brain contain less PKD, further compromising their survival capability. Consistent with this notion, we have demonstrated that PKD1 confers robust neuroprotection against excitotoxicity in excitatory cortical neurons [20]. Other studies have implicated PKD1 in neuroprotection against ischemic neuronal injury through direct phosphorylation of Heat-shock protein 27 (HSP27) [45]. Electrophysiological and biochemical experiments indicate that active PKD1 directly phosphorylates serine residues of NMDARs, leading to their functional inactivation, implying that PKD1 loss-of-function could enhance NMDAR signaling [46]. The observed activation of PKD in the cortex of young R6/1 mice could represent a neuroprotective response aimed at counterbalancing the excess glutamate neurotransmission exerted by cortical glutamatergic afferents on striatal neurons. This aligns with results obtained in cultured excitatory glutamatergic neurons, where NMDAR overstimulation elicited a dual response, initiating with an early peak of PKD activation preceding its inactivation, possibly linked to an unfruitful attempt to stimulate prosurvival pathways [20].

In contrast, NMDA treatment of cultured striatal neurons did not result in net effects in PKD activity within the studied time frame, neither activation nor inactivation by dephosphorylation. This observation highlights distinct responses of these neurons to excitotoxicity compared to cortical excitatory neurons, particularly regarding the stimulation of phosphatases involved in PKD1 inactivation, such as protein phosphatase 1 (PP1). DARPP-32, a marker of MSNs, also known as PP1-regulatory subunit 1B, is a potent inhibitor of this phosphatase [47]. Therefore, the high content of DARPP-32 MSNs may delay the excitotoxic activation of PP1, thus mitigating PKD1 inactivation by dephosphorylation. The regulation of PKD in MSNs might rely more on transcriptional changes than on rapid modulation of its activity by phosphatases. This mechanism may be essential for maintaining PKD1 activity in MSNs, which inherently possess low levels of the kinase and require tight control of its activity for normal function and survival under non-pathological conditions. However, excitotoxicity may eventually overcome this protection by activating calpain, which cleaves DARPP-32, as demonstrated in our study. This degradation step could eventually lead to delayed activation of PP1, contributing to the pathological cascade. Calpain-mediated cleavage of DARPP-32 has been previously shown in Alzheimer´s disease [48], and is likely to occur in various neurological and neurodegenerative diseases with an excitotoxic component, including HD.

Given that NMDAR-mediated excitotoxicity is a pathological hallmark of HD, the early loss of PKD1 in HD striatal neurons may enhance the toxic effects of high glutamate levels. Accordingly, our findings demonstrate that pharmacological inhibition of PKD1 in cultured striatal neurons accelerates DARPP-32 degradation and exacerbates the deleterious effects of excitotoxic NMDAR overstimulation. Furthermore, PKD1 inhibition alone, even in the absence of excitotoxic stimuli, is sufficient to induce apoptotic processes in these cells. Conversely, the expression of active PKD1 confers neuroprotection against excitotoxicity in these neurons and mitigates polyQ-induced apoptosis in an HD cellular model. In clear alignment with in vitro results, intrastriatal ectopic expression of PKD1 prevents the progressive loss of DARPP-32, a hallmark of striatal degeneration in HD. Additionally, the observed trend of increased DARPP-32 expression in areas adjacent to transduced neurons suggests a potential paracrine neuroprotective effect, possibly mediated by the release of neuroprotective trophic factors or signaling molecules. Given that DARPP-32 loss is an early event in HD progression, the ability of PKD1 to preserve its expression points to the kinase as a potential therapeutic target.

While further studies are necessary to elucidate the precise molecular mechanisms underlying PKD1-mediated neuroprotection against mHTT toxicity, our findings strongly support PKD1 as a promising candidate for preventing MSN death associated with HD.

## Conclusion

In summary, our study reveals a substantial reduction in PKD1 levels in neurons of both HD patients and R6/1 mice, and provides compelling evidence for PKD1-mediated neuroprotection against mHTT-induced excitotoxicity and apoptosis. These findings collectively suggest that preserving PKD1 activity in neurons could represent a promising avenue for future therapeutic intervention in HD.

## Materials and methods

### Human brain tissue samples

Human samples came from the Neurological Tissue Bank of the IDIBAPS Biobank (Barcelona, Spain), the Banco de Tejidos Fundación Cien (BT-CIEN, Madrid, Spain) and the Netherlands Brain Bank (Amsterdam, The Netherlands). Written informed consent for brain removal after death for diagnostic and research purposes was obtained from brain donors and/or next of kin. Procedures, information, and consent forms were approved by the Bioethics Subcommittee of Consejo Superior de Investigaciones Científicas (CSIC, Madrid, Spain). When available, the gender, the neuropathological examination of HD cases to assign HD grade from 0–1 to 4 following Vonsattel´s criteria, and the number of CAG repeats are shown in Supplementary Table 1. The *post-mortem* interval in tissue processing was between 2:15 and 23:30 h (Supplementary Table 1).

### Animals

R6/1 transgenic mice for the human exon-1-*HTT* gene [49] in B6CBAF1 background were housed at the Centro de Biología Molecular Severo Ochoa (CBM, CSIC-UAM, Madrid, Spain) animal facility. Male and female 3.5- and 7.5-month-old R6/1 or WT littermates were employed. Wistar rat embryos of 19 days were obtained from the animal care facility at the Instituto de Investigaciones Biomédicas Sols-Morreale (IIBm, CSIC-UAM, Madrid, Spain). All animals were housed maximum four per cage with food and water available *ad libitum* and maintained in a temperature-controlled environment on a 12/12 h light–dark cycle with light onset at 07:00 h. Animal housing and maintenance protocols followed the guidelines of Council of Europe Convention ETS123, recently revised as indicated in the Directive 86/609/EEC. Animal experiments were performed under protocols approved by institutional (IIBm, CBM, and CSIC) and local Ethical Committees and were conformed to the appropriate national legislations (RD 53/2013).

### Primary culture, transfection, and treatment of striatal neurons

Striatal neurons were prepared from 19-day-old Wistar rat embryos [50]. Briefly, after removal of meninges a portion of the anterior cortex was cut and the striatal tissue was dissected and incubated with trypsin solution (Thermo Fisher Scientific, Waltham, MA, USA) for 15 min at 37 °C. After two washes with Hank´s Balance Salt Solution (HBSS) with 1 mM HEPES, tissue was mechanically dissociated in neuronal plating media [Minimum essential medium (MEM) supplemented with penicillin (100 U/mL) streptomycin (100 U/mL), 22.2 mM glucose, Glutamax-I, 5% fetal bovine serum and 5% horse serum, all from Thermo Fisher Scientific]. Cells were plated at a density of 2.5 × 10^5^ or 5 × 10^5^ cells per well of 24- or 12-multiwell plates, respectively, on glass coverslips or directly the plate covered with poly-L-lysine (10 μg/ml) and laminin (4 μg/ml) (both from Merck/Sigma-Aldrich, Darmstadt, Germany). Approximately 3 h after plating, HBSS was replaced with neuronal growth media (Neurobasal medium supplemented with B27 and Glutamax-I from Thermo Fisher Scientific). At 3 and 7 days in vitro (DIV) one sixth of the media was replaced with new neuronal growth media. Neurons were incubated at 37 °C, 5% CO_2_ and 95% humidity and used at DIV9. Depending on the experiment, neurons were treated for different time periods with: 50 μM NMDA and 10 μM glycine (both from Merck/Sigma-Aldrich), 5 μM PKD-specific pharmacological inhibitor CRT0066101 (CRT; Abcam, Cambridge, UK), 20 μM calpain inhibitor (Ci-III; Merck/Calbiochem), 200 μM DL-2-amino-5-phosphonopentanoic acid (DL-AP5; Tocris Bioscience, Bristol, UK) or 2 mM EGTA (Merck/Sigma-Aldrich). Excitotoxicity was induced by overstimulation of NMDAR with the co-agonists NMDA and glycine (a treatment referred as “NMDA”) at the concentrations indicated above. Inhibitors were added 1 h before NMDA-treatment and remained in the culture media for the duration of the experiment.

Primary striatal neurons DIV6 were transfected with Lipofectamine 2000 reagent in Neurobasal Medium (both from Thermo Fisher Scientific) according to the manufacturer’s specifications. After 2 h, liposomes were removed, and neurons were fed with neuronal conditioned medium and maintained in culture for 3 days (DIV9) before experimental treatments. Two plasmids were used for the neurospecific expression driven by the human synapsin (h*SYN*) promoter of GFP only (Control) or constitutively active PKD1 (the quadruple mutant PKD1-Y^93^E/Y^469^E/S^744^E/S^748^E) fused to GFP (PKD1-Ca) [20].

### N2a maintenance and transfection

Neuro-2a neuroblastoma (N2a) cells, obtained from ATCC (CCL-131; LGC Standards S.L.U., Barcelona, Spain), were cultured at 37 °C, 5% CO_2_ and 95% humidity in Dulbecco’s Modified Eagle Medium (DMEM) completed with 10% fetal bovine serum, penicillin (100 U/mL), streptomycin (100 U/mL) and glutamine (2 mM), all these reagents from Thermo Fisher Scientific. The day before transfection 6 × 10^4^ cells per condition were placed in a 24-well plate with glass coverslips and allowed to attach to the plate. We used constructs expressing CFP-tagged human *HTT* exon 1 harboring a pathogenic polyQ tract of 94 CAG repeats (PolyQ94) or a normal, non-pathogenic polyQ tract of 16 CAG repeats (PolyQ16). N2a cells were co-transfected with Lipofectamine 2000 reagent and the following plasmids combinations: PolyQ16 and p-EF-BOS-GFP (hereafter referred as Control); PolyQ94 and GFP; PolyQ16 and p-EF-BOS-PKD1-Ca (hereafter referred as PKD1-Ca); PolyQ94 and PKD1-Ca. PolyQ16, PolyQ94 [51], GFP and PKD1-Ca [20] plasmids have been previously described. Cells were used for subsequent assays 24 h after transfection.

### Annexin V-FITC and Propidium iodide cell death assay in adherent cultured striatal neurons

The Annexin V-FITC (AxV)/Propidium iodide (PI) detection kit (ThermoFisher Scientific, V13242) was used and optimized to measure apoptosis and necrosis in DIV10 striatal neurons grown on coverslips. Following incubation with NMDA and CRT for various time points, neurons were washed with PBS and incubated with AxV and PI (diluted 1:20 and 1:50, respectively) in binding buffer (BB) (10 mM HEPES, 150 mM NaCl and 2.5 mM CaCl_2_) for 10 min at room temperature (RT) in the dark, according to the manufacturer’s instructions. After staining, neurons were washed with cold BB, fixed in 1% formaldehyde prepared in cold BB for 10 min at 4 °C, and then washed again with PBS. Labeled neurons were immediately imaged using a fluorescence microscope (Nikon, Tokyo, Japan).

### Lentiviral production and transduction of cultured striatal neurons

Lentiviral suspensions for GFP and GFP-PKD1-Ca expression were produced in HEK293T cells, obtained from ATCC (CRL-1573; LGC Standards S.L.U., Barcelona, Spain), as previously described [20]. Briefly, HEK293T cells were transfected with lentiviral and packaging vectors using Lipofectamine 2000 reagent and OPTI-MEM medium (ThermoFisher Scientific) for 4 h, following the manufacturer’s instructions. The medium was then replaced with IMDM-GlutaMAX Supplement complemented with 10% fetal bovine serum, penicillin (100 U per ml), and streptomycin (100 U per ml). Supernatants were collected after 48 h. To concentrate viral particles, the suspension was filtered using a Steriflip-HV 0.22-μm filter unit (Millipore, Billerica, MA, USA) and ultracentrifuged at 26,000 rpm for 2 h at 4 °C in a SW28 Beckman Coulter rotor. Viral pellets were resuspended in cold PBS and stored at −80 °C. Primary striatal neurons at DIV3 were transduced with the concentrated lentiviral suspensions directly added to the growth medium, allowing for expression over the next 7 days.

### Stereotaxic intracerebral injection of lentiviral particles

Male 32-week-old R6/1 mice were anesthetized with isoflurane and secured in a stereotaxic frame. Lentiviral particles encoding GFP-PKD1-Ca were stereotaxically injected into the striatum using a 5 μl-capacity syringe fitted with a 33-gauge beveled needle (Neuros Syringe, Model 75 RN, Hamilton Iberia, Barcelona) [52]. The injection coordinates (in mm) relative to bregma in the antero-posterior (A-P), medio-lateral (M-L), and dorso-ventral (D-V) axes were as follows: 0.6 A-P; +1.20 M-L and 3.0 D-V. A total volume of 1 μl of viral suspension was infused at a rate of 0.2 μl per min. Mice were anesthetized and perfused for brain fixation and further immunofluorescence analysis 25 days post-injection.

### Antibodies

Rabbit polyclonal antibodies used are the following: total PKD (PKD_T_), (Thermo Fisher Scientific); phospho-PKD-S^916^, cleaved caspase-3 (AC3) and dopamine- and cAMP-regulated phosphoprotein of 32 kDa (DARPP-32; Cell Signaling Technology, Danvers, MA, USA); neuronal specific enolase (NSE; Merck/Millipore); Poly(ADP-ribose) polymerase-1 (PARP-1, Santa Cruz Biotechnology, CA, USA). Mouse monoclonal antibodies used are: β-actin and spectrin (Merck/Sigma-Aldrich); DARPP-32 (BD Biosciences, Franklin Lakes, NJ, USA); NeuN (Merck/Millipore). Goat polyclonal antibody recognizing glial fibrillary acidic protein (GFAP) and chicken polyclonal antibody against microtubule associated protein 2 (MAP2) were from Abcam. Secondary antibodies used are the following: horseradish peroxidase-conjugated anti-rabbit and anti-mouse (Santa Cruz Biotechnology); donkey anti-rabbit, anti-mouse, anti-goat and anti-chicken conjugated with Alexa Fluor 488, 546, 555 or 647 (Thermo Fisher Scientific). Detailed information about all antibodies and dilutions used for the different applications is given in Supplementary Table 2.

### Preparation of protein extracts and immunoblot analysis

Samples from human brain were stored at −80 °C and ground with a mortar using liquid nitrogen to prevent thawing of the samples. Mouse brains were quickly dissected on an ice-cold plate and the different structures stored at −80 °C. Extracts from human brain cortex were prepared in 1% SDS in PBS, 5 mM EDTA and 5 mM EGTA, containing protease and phosphatase inhibitors. Samples homogenization was performed with a POLYTRON System PT 1200 E (Kinematica AG, Lucerne, Switzerland) and lysates were then centrifuged at 21,000 *g* for 30 min at 4 °C.

Mouse protein extracts were prepared by homogenizing samples in ice-cold extraction buffer [20 mM HEPES pH 7.4, 100 mM NaCl, 20 mM NaF, 1% Triton X-100, 1 mM sodium orthovanadate, 1 μM okadaic acid, 5 mM sodium pyrophosphate, 30 mM β-glycerophosphate, 5 mM EDTA, protease inhibitors (cOmplete^TM^, Merck/Roche)]. Homogenates were centrifuged at 15,000 rpm for 15 min at 4 °C.

To obtain protein extracts from primary cultures cellular lysis was performed using RIPA buffer (25 mM Tris-HCl, pH 7.6, 1% Triton X-100, 0.5% sodium deoxycholate, 0.1% SDS, 150 mM NaCl and 1 mM DTT) with protease and phosphatase inhibitors (1 mM phenylmethylsulfonyl fluoride, 10 μg/ml aprotinin, 10 μg/ml leupeptin, 50 mM sodium fluoride, 5 mM sodium pyrophosphate and 1 mM sodium metavanadate, all from Merck/Sigma-Aldrich) added to the cultures, maintained in agitation for 30 min at 4 °C and subsequent centrifugation at 21,900*xg* for 30 min at 4 °C. Protein content in all extracts was determined by BCA assay (Thermo Fisher Scientific).

Proteins were resolved in 8% SDS-PAGE gels and transferred to nitrocellulose membranes, blocked for 30 min at RT with 5% non-fat dried milk in tween-Tris-buffered saline (TBS-T; 137 mM NaCl, 20 mM Tris-HCl pH 7.6, 0.05% Tween-20) and incubated overnight at 4 °C with primary antibodies. Blots were then washed in TBS-T and incubated for 1 h at RT with horseradish peroxidase-conjugated secondary antibodies. Protein bands were detected by ECL chemiluminescence (Perkin-Elmer Life Sciences, Waltham, MA, USA). Immunoblot signals were quantified by densitometric analysis (NIH ImageJ, Bethesda, MD, USA), normalized using β-actin or NSE intensities, and expressed relative to values obtained in their respective controls. The doublet obtained using phospho-PKD-S916 was quantified and represented in the graphs as total p-PKD (S^916^) signal. Immunoblot images have been cropped for presentation. Full-size, uncropped immunoblot images are provided in the Supplemental Material.

#### RNA isolation, reverse transcription, and quantitative real-time PCR (RT-qPCR)

Total tissue RNA was extracted from striatum and cortex of HD and control subjects and from striatum of R6/1 and WT mice using the Maxwell® 16 LEV simplyRNA Tissue Kit (Promega, Madison, WI, USA). The resulting total RNA (750 ng) was used for cDNA synthesis with a Super Script III First-Strand Synthesis SuperMix kit from Thermo Fisher Scientific (PN 11752250) with the amplification protocol 30″ at 95 °C + (5″ at 95°C + 5″ at 60 °C) × 40 + (5″ at 60°C + 5″ at 95 °C). Quantification was performed by real-time PCR using a CFX 384 System (Bio-Rad, Hercules, CA, USA) in combination with SsoFast Eva Green (Bio-Rad), as per manufacturer’s protocol and 1 μl of primer pair was used. Data were analyzed by GenEx 5.3.7 software (Multid AnaLyses AB, Göteborg, Sweden). The mRNA levels were normalized first relative to total RNA and then relative to the mean of GADPH, β-actin and β-tubulin gene expression for human and the mean of β-actin and β-tubulin or the mean of 18S ribosome subunit, GADPH, β-actin and β-tubulin gene expression for 3.5- or 7.5-month-old mouse, respectively, in each sample. mRNA abundance was calculated using the 2^-ΔΔCt^ method and assigned arbitrarily 1 value to one control sample. The primers used are indicated in Supplementary Table 3. The qPCR/NGS experimental design/follow up/data analysis/service was provided by the Genomics and NGS Core Facility at the CBM (CSIC-UAM) which is part of the CEI UAM + CSIC, Madrid, Spain - http://www.cbm.uam.es/genomica.

### Tissue preparation, immunofluorescence, and immunohistochemistry

Mice euthanasia was performed using CO_2_. Brains were immediately removed and dissected on an ice-cold plate and left hemispheres, processed for histology, placed in 4% paraformaldehyde in Sorensen’s phosphate buffer overnight and then immersed in 30% sucrose in PBS for 72 h. Once cryoprotected, samples were included in optimum cutting temperature compound (OCT; Tissue-Tek, Sakura Finetek Europe, Alphen aan den Rijn, The Netherlands), frozen and stored at −80 °C before use. Sagittal sections 30 μm thick were cut on a cryostat (Thermo Fisher Scientific), collected, and stored free floating in glycol containing buffer (30% glycerol, 30% ethylene glycol in 0.02 M phosphate buffer) at −20 °C. For human samples analysis, formalin-fixed (4%, 24 h), paraffin embedded sections (5-μm thick) from cortex and striatum mounted on superfrost-plus tissue slides (Menzel-Gläser, Braunschweig, Germany) were first deparaffinized. Next, peroxidase activity was quenched with 0.3% H_2_O_2_ in methanol for 30 min, followed by antigen retrieval with 10 mM pH 6.0 citrate buffer heated by microwave for 15 min.

For immunohistochemistry analysis 5-μm thick formalin-fixed (4%, 24 h), paraffin-embedded sections from human cortex and striatum were mounted onto slides and used. After deparaffination, endogenous peroxidase activity quenching was performed by immersion in 0.3% H_2_O_2_ in methanol for 30 min and antigen retrieval in 10 mM pH 6.0 citrate buffer heated by microwave for 15 min was done. In mouse brain floating sections, endogenous peroxidase was inactivated by incubating sections in a solution of 0.3% H_2_O_2_ in PBS for 45 min. Next, human or mouse brain sections were pretreated for 1 h with blocking solution consisting in 1% bovine serum albumin, 0.5% fetal bovine serum, and 0.3% Triton X-100 in PBS, and subsequently incubated over-night with PKD_T_ or p-PKD (S^916^) antibody in blocking solution. After washing primary antibody with PBS, sections were incubated with anti-rabbit secondary biotin-conjugated antibody for 2 h. Finally, chromogenic signal was amplified by incubating samples for 1.5 h with avidin–biotin complex using Elite Vectastain kit (Vector Laboratories, Burligame, CA, USA). Chromogen 3-3ʹ diaminobenzidine (DAB) reactions were performed as manufacturer’s protocol with SIGMA-FAST (Merck/Sigma) for 10 min. Next, mice sections were mounted with Mowiol (Merck/Sigma) while human where first dehydrated and then mounted with DePex (SERVA, OK, USA).

For immunofluorescence analysis, floating parasagittal sections from WT and R6/1 mice were incubated in blocking solution with primary antibodies overnight at 4 °C and subsequently incubated 2 h at 4 °C with fluorescent-labeled secondary antibodies. Then, slices were rinsed in PBS and nuclei were stained with DAPI and mounted with Fluoromount G^®^ (SouthernBiotech, Birmingham, AL, USA).

Floating human postmortem striatum 50-μm sections were treated with citrate buffer (pH 6.0) for antigen retrieval and incubated in 10% donkey serum for 1 h. For triple immunofluorescence analysis, sections were incubated with primary antibodies for 2 days in blocking solution, followed by washes in PBS and incubation with secondary antibodies in the same solution. Then, slices were rinsed in PBS and nuclei were stained with DAPI. In addition, human sections were treated with a saturated solution of Sudan black B (Merck) for 15 min to block autofluorescence of lipofuscin granules, washed and mounted with Fluoromount G^®^ (SouthernBiotech).

Primary neurons or N2a cells grown on coverslips were fixed with 4% PFA containing 4% sucrose for 20 min, permeabilized with 0.1% Triton X-100 in PBS for 5 min and then incubated in 4% bovine serum albumin in PBS for 30 min. Coverslips were then incubated for 1 h at RT or overnight at 4 °C with the appropriate primary antibody in blocking solution followed by the corresponding secondary antibody. Nuclei were stained with DAPI or TO-PRO^TM^-3 Iodide (642/661) (Thermo Fisher Scientific) and coverslips were mounted with Fluoromount G^®^ (SouthernBiotech).

### Quantitative analysis

Chromogen intensity in brain sections was quantified using the reciprocal intensity (r) method [53], where: *r* = 255−y, 255 being the maximum pixel intensity of unstained area (as measured by the mean gray value with ImageJ software) and y being the mean intensity of the region of interest (ROI) randomly selected in the cerebral cortex. Reciprocal intensity was quantified from five ROIs per field of individual sections from three mice per group. For each field, background measurements from a free-cell zone were subtracted. To quantify PKD_T_ immunofluorescence signal in NeuN positive cells in brain sections from control, HD patients, WT, or R6/1 mice, the intensity of PKD_T_ staining was measured in 17-85 NeuN positive neurons per human sample or 24–94 NeuN positive neurons per mouse sample, and compared to the mean value obtained from control individuals or WT animals, respectively. For quantifying PKD_T_ immunofluorescence signal in GFAP-positive cells in brain sections from control or HD patients, the intensity of PKD_T_ staining was measured in the soma of 21 or 58 GFAP-positive striatal astrocytes in control or HD samples, respectively. The number of PKD_T_ positive events with astrocytic-like morphology in the globus pallidum was calculated as the number of positive events per area where events were counted (*n* = 20–148 events per mice). Fluorescence microscopy image analysis to quantify apoptotic striatal neurons in culture was performed using ImageJ software. AxV^+^/PI^−^ neurons were considered early apoptotic cells, AxV^+^/PI^+^ were considered necrotic and late apoptotic cells, while AxV^−^/PI^−^ cells were considered living cells. The percentage of apoptotic neurons was expressed as AxV^+^/PI^−^ neurons relative to the total number of neurons in each condition. To measure the percentage of area covered by MAP2 staining in striatal cultured neurons, a z-scale projection of 4 confocal images using a tile scale covering an area of 1417 × 1417 µm was used. The same threshold was applied to every projection across different experimental groups. To quantify the percentage of neurons that were DARPP-32 positive relative to the total number of transfected (GFP positive) neurons, neurons were considered DARPP-32 positive when the staining intensity of this marker in NeuN-positive cells exceeded 5% of the total area of each individual cell body. In striatal neuronal cultures transduced with lentivirus for GFP and GFP-PKD1-Ca expression, we quantified the percentage of neurons that were DARPP-32 positive relative to the total number of transduced neurons (GFP positive). Neurons were considered DARPP-32 positive after setting a threshold to remove background and non-specific staining. The same threshold was applied to all projection across experimental conditions. The percentage of neurons that were GFP positive and exhibited nuclear condensation was quantified in NMDA untreated or treated cultures, and represented as the percentage of nuclear condensation relative to total GFP neurons. N2a apoptotic cell death was determined by quantifying cleaved-caspase 3-positive cells relative to the total number of transfected cells counted (*n* = 500–1500) in maximum intensity projections of confocal microscopy serial sections corresponding to the different experimental conditions. Cells were considered transfected when positive for both reporters CFP and GFP, indicative of double transfection with HTT and PKD1 constructs, respectively. For quantifying DARPP-32 immunofluorescence signal in brain sections from R6/1 mice injected with GFP-PKD1-Ca lentiviral particles into the striatum, the intensity of DARPP-32 staining was measured in an ROI occupied by neurons transduced with GFP-PKD1-Ca in the left-brain hemisphere using a densitometric thresholding technique implemented with ImageJ 1.47 d software (NIH). The same threshold was applied to measure the intensity of DARPP-32 in equivalent ipsilateral or contralateral ROIs, including non-expressing GFP-PKD1-Ca neurons located in the same left hemisphere or in the right contralateral hemisphere.

### Statistical analysis

Statistical analysis was performed with GraphPad Prism (version 5.00 for Windows, GraphPad Software, San Diego, CA, USA). Appropriate tests were chosen according to data distribution. Differences were analyzed by one-way analysis of variance (ANOVA) with Bonferroni post-test or Student *t* test, as appropriate. Differences were considered significant at *P* < 0.05. Data are represented as mean ± s.e.m. derived from the indicated number of patients or non-affected individuals, mice, or experiments. This number is shown in each figure as dots and is specified in the corresponding figure legends. All experiments were carried out with at least three biological replicates. For two-group comparison, data were analysed with two-tailed unpaired Student´s *t* test. For multiple comparisons, One-way ANOVA with a Bonferroni post-hoc test was used. A critical value for significance of *P < 0.05, **P < 0.01 or ***P < 0.001 was applied throughout the study. Investigators were not blinded to allocation during the experiments, use of animals or outcome assessment. For animal studies and experiments with animal derived tissues and cells no randomization was used, and no statistical method was used to predetermine sample size. Sample size was chosen empirically based on our previous experience in the calculation of experimental variability.

## Supplementary information

Supplementary File

Uncropped WB

## Data Availability

The datasets generated and/or analysed during the current study are available from the corresponding authors on reasonable request.
