# Supplementary material for: Down-regulation of neuroprotective protein kinase D in Huntington´s disease
Source: Cell Death Dis. 2025 Jun 3;16(1):418. doi: 10.1038/s41419-025-07688-9 (PMC12134097; doi:10.1038/s41419-025-07688-9)
Supplement: Supplementary file 1 — Supplementary File [file 41419_2025_7688_MOESM1_ESM.pdf]

SUPPLEMENTARY INFORMATION

DOWN-REGULATION OF NEUROPROTECTIVE PROTEIN KINASE D IN HUNTINGTON´S DISEASE

Álvaro Sebastián-Serrano[1,2,3,#,*], Ana Simón-García[1,2,#], María Santos-Galindo[2,4], Marina Prudencio Sánchez-Carralero[1,2], Alberto H.-Alcántara[5], Cristina Clemente[5], Julia Pose-Utrilla[2,4], Miguel R. Campanero[5,6], Eva Porlan[2,4,7,8,9], José J. Lucas[2,4] and Teresa Iglesias[1,2,*]

[1]Instituto de Investigaciones Biomédicas Sols-Morreale. Consejo Superior de Investigaciones Científicas-Universidad Autónoma de Madrid (CSIC-UAM). C/ Arturo Duperier, 4. 28029 Madrid, Spain.
[2]Centro de Investigación Biomédica en Red de Enfermedades Neurodegenerativas (CIBERNED), Instituto de Salud Carlos III, Avda Monforte de Lemos, 3-5. 28029 Madrid, Spain.
[3]Department of Biochemistry and Molecular Biology, Medical School, Complutense University of Madrid, Plaza Ramón y Cajal, S/N, 28040 Madrid, Spain.
[4]Molecular Neuropathology Unit, Centro de Biología Molecular Severo Ochoa (CSIC-UAM), C/ Nicolás Cabrera 1, 28049 Madrid, Spain.
[5]Tissue and Organ Homeostasis Program, Centro de Biología Molecular Severo Ochoa (CSIC-UAM), C/ Nicolás Cabrera 1, 28049 Madrid, Spain.
[6]Centro de Investigación Biomédica en Red de Enfermedades Cardiovasculares (CIBERCV), Instituto de Salud Carlos III, Avda Monforte de Lemos, 3-5. 28029 Madrid, Spain.
[7]Departamento de Biología Molecular, Universidad Autónoma de Madrid, C/ Francisco Tomás y Valiente, 7, Ciudad Universitaria de Cantoblanco, 28049 Madrid (Spain).
[8]Instituto Universitario de Biología Molecular – UAM, C/ Nicolás Cabrera, 1, 28049 Madrid, Spain.
[9]Instituto de Investigación Sanitaria del Hospital Universitario La Paz (IdiPAZ), Instituto de Salud Carlos III, Av. Monforte de Lemos, 3-5. Pabellón 11. Planta 0 28029 Madrid, Spain.
[#]These authors contributed equally: Álvaro Sebastián-Serrano, Ana Simón-García.

*Correspondence to: Teresa Iglesias, E-mail: tiglesias@iib.uam.es; Álvaro Sebastián-Serrano, E-mail: alvarseb@ucm.es

This PDF file includes:

Figures S1 to S7

Tables S1 to S3

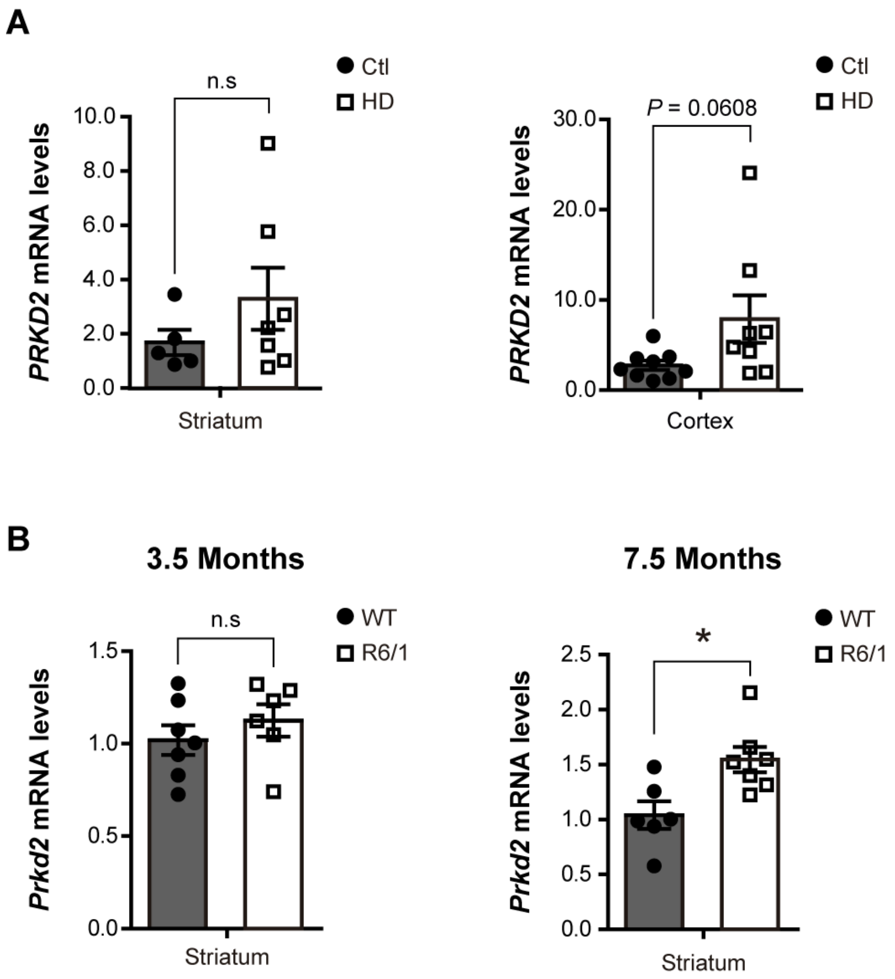

Supplementary Figure 1. Increased striatal PKD2 mRNA in the R6/1 mouse model, but not in HD human samples. A. qRT-PCR analysis of *PRKD2* mRNA from striatum and cortex of HD patients (n = 7-8) and non-affected individuals (n = 5-9). **B.** qRT-PCR analysis of *Prkd2* mRNA from striatum of 3.5-month-old or 7.5-month-old R6/1 (n = 6-7) and WT (n = 6-7) mice (P = 0.0126). Data are represented as mean ± SEM. * $P < 0.05$ or n.s (not significant) using unpaired Student's t test.

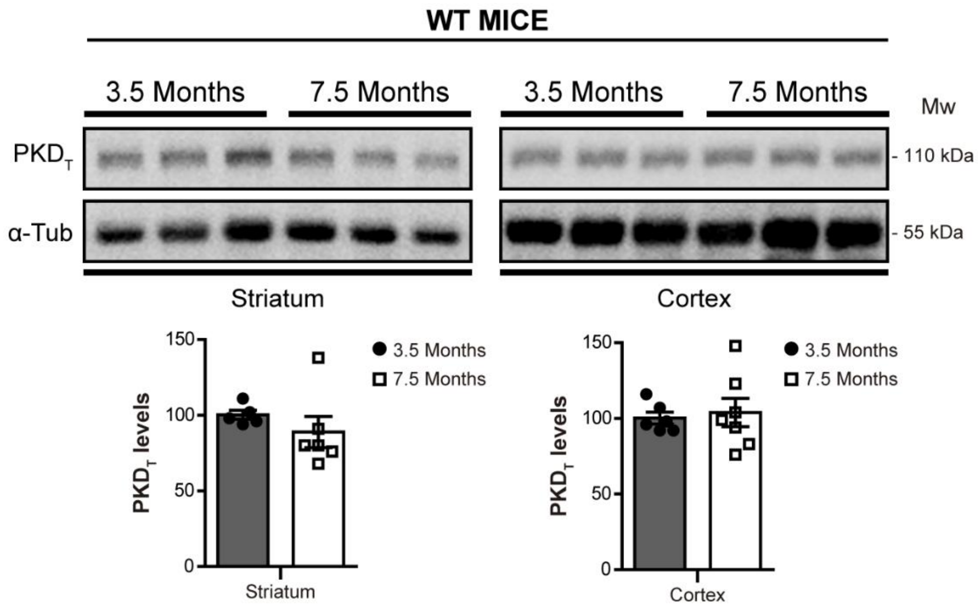

Supplementary Figure 2. The striatum and cortex from 3.5- and 7.5-month-old wild type mice do not show changes in PKD protein levels. Representative immunoblots of PKD_T and quantification of protein levels in homogenates from striatum and cerebral cortex of 3.5- (n = 5-6) and 7.5-month-old (n = 6-7) wild type (WT) mice. Levels of α-tubulin (α-Tub) were used as loading control for normalization purposes. Data are represented as mean ± SEM. Non-significant differences were found using unpaired Student's t test.

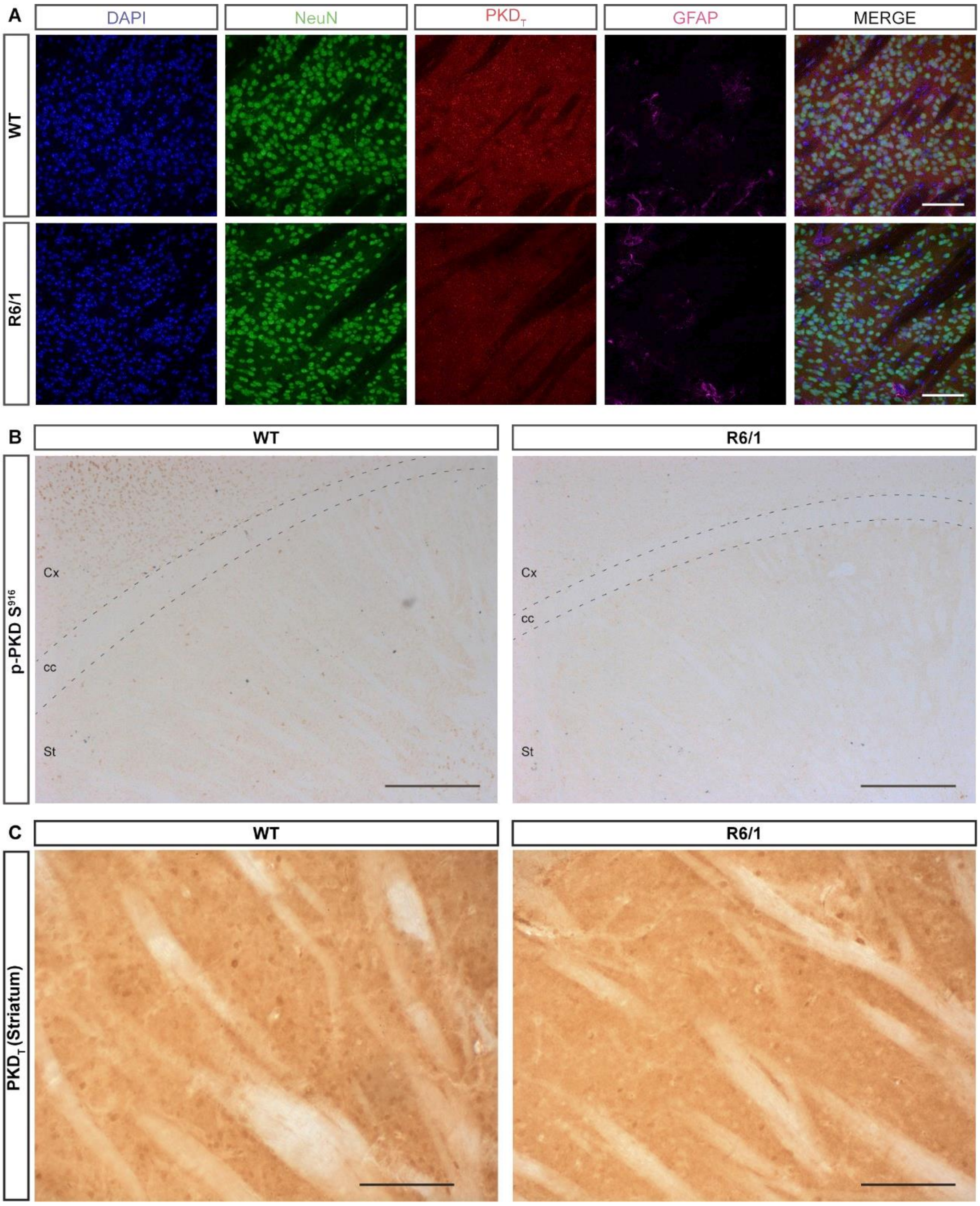

Supplementary Figure 3. Overall decrease of striatal PKD immunostaining signal in the R6/1 mouse model. A. Representative confocal microscopy images at low magnification of PKD$_T$ (red channel) in neurons (NeuN$^+$, green channel) or astrocytes (GFAP$^+$, purple channel) in striatum of 7.5-month-old WT and R6/1 mice. Nuclei were stained with DAPI. Scale bar: 100 µm. **B.** p-PKD (S^{916}) immunohistochemistry in cerebral cortex (Cx), corpus callosum (cc) and striatum (St) in brain sections from 7.5-month-old WT and R6/1 mice. Scale bar: 500 µm. **C.** PKD$_T$ immunohistochemistry in striatum from 7.5-month-old WT and R6/1 mice. Scale bar: 50 µm.

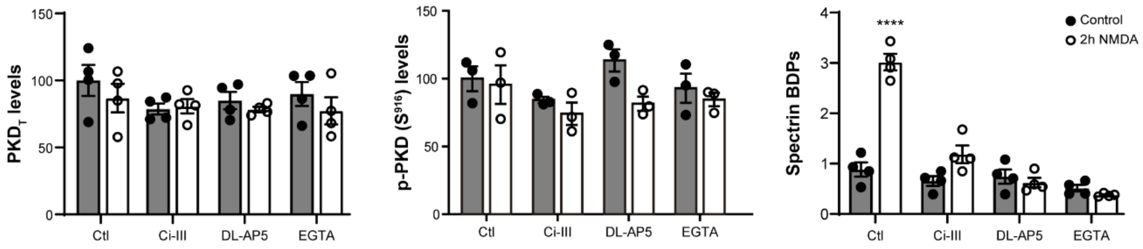

Supplementary Figure 4. Pretreatment of cultured rat primary striatal neurons with the calpain inhibitor Ci-III, the NMDAR antagonist DL-AP5 or the calcium chelator EGTA blocks excitotoxicity without altering PKD levels or activity. Graphs represent the quantification of PKD_T, p-PKD (S^{916}) and spectrin BDPs immunoblot signal relative to loading control NSE of cultured striatal neurons pre-treated for 1 h with the calpain inhibitor Ci-III, the NMDAR antagonist DL-AP5, or the Ca^{2+} chelator EGTA and then stimulated with NMDA for 2 h. n = 3 or 4 independent experiments. All data are represented as mean ± SEM. **** $P < 0.0001$ using one-way ANOVA, followed by Bonferroni's post hoc test. Representative immunoblot images are in Figure 4D.

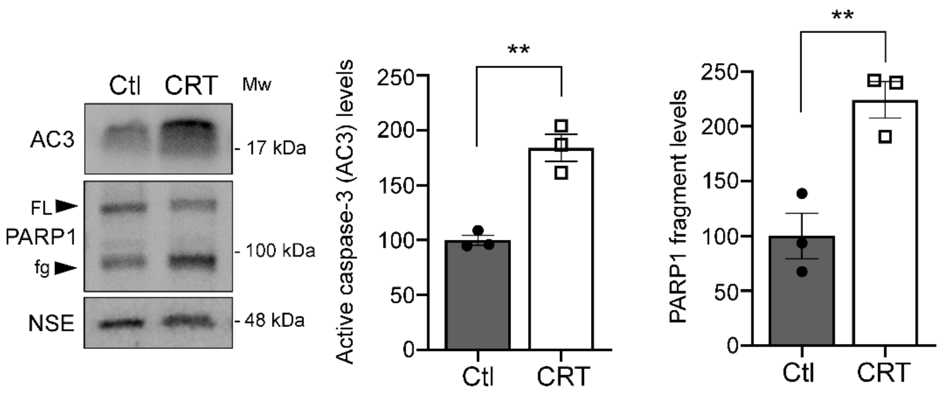

Supplementary Figure 5. CRT induces apoptosis in cultured striatal neurons. Representative immunoblots of active caspase-3 (AC3) and PARP-1 full-length (FL) and its cleaved fragment (fg) of lysates from cultured striatal neurons treated with the PKD-specific pharmacological inhibitor CRT0066101 (CRT, 5 µM) up to 48h. Graphs represent the quantification of AC3 and PARP-1 fg immunoblot signal relative to loading control NSE. n = 3 independent experiments. Data are represented as mean ± SEM. ** $P < 0.01$, using unpaired Student's *t* test.

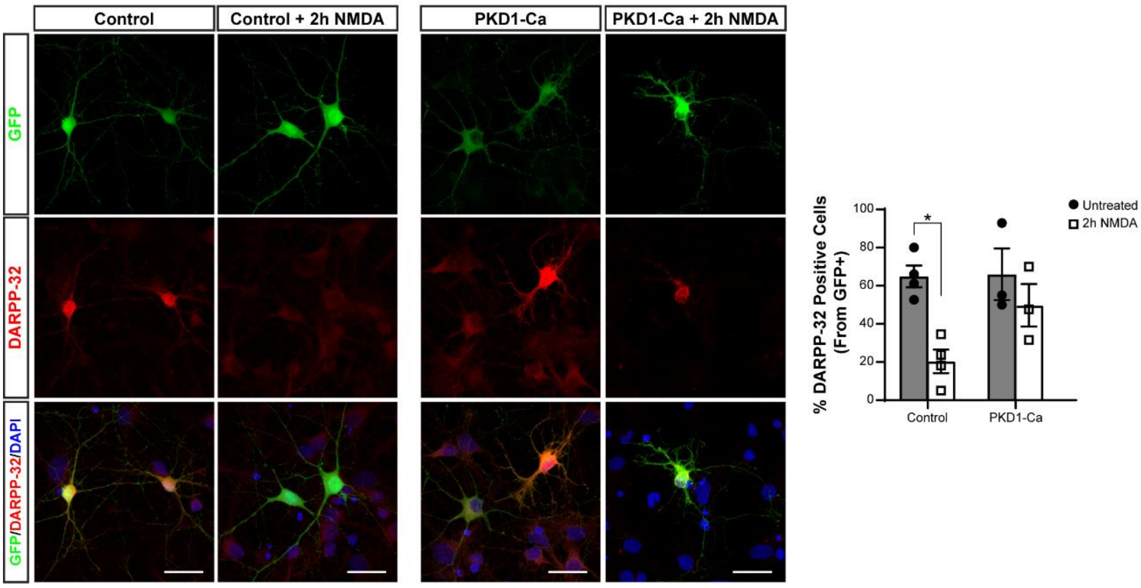

Supplementary Figure 6. Transfection of PKD1 constitutive active is sufficient to confer neuroprotection to cultured striatal neurons against NMDA-induced excitotoxicity. Representative confocal microscopy images of GFP, DARPP-32 and DAPI signal of GFP- (Control-) or PKD1-Ca-transfected neurons treated or not with NMDA for 2 hours. Scale bar: 25 µm. Graph represents the percentage of DARPP-32$^+$ surviving neurons after NMDA treatment, relative to total GFP$^+$ neurons in untreated conditions (n = 124-159 neurons per condition; n = 3-4 individual experiment per group). All data are represented as mean ± SEM. * $P < 0.05$, using one-way ANOVA, followed by Bonferroni's post hoc test.

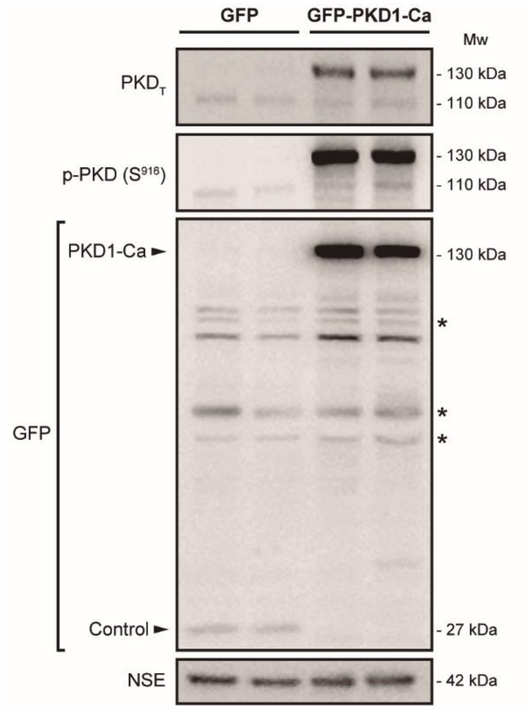

Supplementary Figure 7. Elevated expression and activity of PKD1-Ca in lentiviral transduced cultured striatal neurons. Representative immunoblots of PKD$_T$, p-PKD (S^{916}) and GFP in homogenates from cultured primary mature striatal neurons transduced with lentiviral particles containing GFP or GFP-PKD1-Ca. Levels of NSE were used as loading control.

* Unspecific bands.

Supplementary Table 1. Case information for HD and control cases

Diagnosis	Vonsattel grade	Sex	Age (years)	PMI	Structure analyzed	Experiment
HD	n/a	F	28	4:15	Cx	qPCR
HD	n/a	M	59	5:30	Cx	qPCR
HD	4	F	64	5:00	St	qPCR
HD	n/a	F	67	6:05	St	qPCR
HD	n/a	M	44	n/a	St	WB
HD	n/a	M	43	n/a	St	WB
HD	n/a	F	68	n/a	St	WB
HD	46, g. 3	M	57	7:30	St	qPCR
HD	47, g. 2-3	F	50	5:40	St	qPCR
HD	n/a	M	71	10:15	St, Cx	qPCR, WB
HD	n/a	F	65	15:15	St, Cx	qPCR, WB
HD	n/a	M	68	4:00	St, Cx	qPCR, WB
HD	n/a	M	60	13:05	Cx	qPCR, WB
HD	n/a	F	72	7:00	Cx	qPCR, WB
HD	n/a	F	72	17:00	Cx	qPCR, WB
HD	40, g. 3	F	85	5:30	St	IF
HD	g. 3-4	M	55	7:00	St	IF
HD	48, g. 3	F	55	15:00	St	IF
HD	n/a	M	72	n/a	St, Cx	IHC
HD	n/a	M	70	n/a	St, Cx	IHC
HD	g. 3	M	72	n/a	St, Cx	IHC
HD	41, g.4	M	61	7:00	Cx	IHC
Control	-	M	68	8	St	WB
Control	-	M	56	n/a	St	WB
Control	-	F	58	n/a	St	WB
Control	-	M	55	7:30	St	qPCR
Control	-	M	76	6:45	St	qPCR
Control	-	n/a	n/a	n/a	St, Cx	qPCR, WB
Control	-	n/a	n/a	n/a	St, Cx	qPCR, WB
Control	-	n/a	n/a	n/a	St	qPCR, WB
Control	-	M	85	5:45	Cx	WB
Control	-	F	71	8:30	Cx	qPCR, WB
Control	-	F	81	4:00	Cx	qPCR, WB
Control	-	F	64	5:00	Cx	qPCR, WB
Control	-	M	78	2:15	Cx	qPCR
Control	-	M	71	12:00	Cx	qPCR
Control	-	F	78	3:40	Cx	qPCR
Control	-	M	60	8:10	Cx	qPCR
Control	-	M	78	6:00	St	IF
Control	-	M	64	10:00	St	IF
Control	-	F	64	23:30	St	IF
Control	-	M	58	14:00	St, Cx	IHC
Control	-	M	78	4:00	St, Cx	IHC
Control	-	M	78	12:00	St, Cx	IHC
Control	-	F	74	-	Cx	IHC

n/a, not available. M, male. F, female. PMI, *post-mortem* interval (h). St, Striatum. Cx, Cerebral Cortex. IF: immunofluorescence, IHQ: immunohistochemistry, qPCR: quantitative PCR, WB: Western blot

Supplementary Table 2. Details of the antibodies used.

**Antibody**	**Supplier**	**Catalog number**	**Dilution**
β-actin	Sigma-Aldrich	A5441	WB 1:5,000
Cleaved Caspase-3 (AC3)	Cell Signaling Technology	9661	IMF 1:50*<br>WB 1:250*
DARPP-32	Cell Signaling Technology	2302	IMF 1:500*<br>WB 1:1,000*
DARPP-32	BD Biosciences	61520	IMF 1:500*
GFAP	Abcam	ab53554	IMF 1:2,000* 1:500**<br>IHC 1:2,000*
Microtubule associated protein 2 (MAP2)	Abcam	ab5392	IMF 1:10,000*
NeuN, clone A60	Millipore	MAB377	IMF 1:1,000* 1:100**
Neuronal specific enolase (NSE)	Millipore	AB951	WB 1:10,000*
PARP-1	Santa Cruz Biotechnology	sc-7150	WB 1:2,000*
PKD_T	Thermo Fisher Scientific	PA578126	IHC 1:500* 1:100**<br>IMF 1:500* 1:100**<br>WB 1:1,000* 1:500**
phospho-PKD-S^{916}	Cell Signaling Technology	2051	WB 1:250*<br>IMF 1:250*
Spectrin	Millipore	MAB1622	WB 1:2,000*
HRP anti-Rabbit	Santa Cruz Biotechnology	sc-2004	WB 1:5,000
HRP anti-Mouse	Santa Cruz Biotechnology	sc-2005	WB 1:2,000
Donkey anti-Rabbit 555	Thermo Fisher Scientific	A31572	IMF 1:500* 1:200**
Donkey anti-Mouse 488	Thermo Fisher Scientific	A21202	IMF 1:500* 1:200**
Donkey anti-Mouse 546	Thermo Fisher Scientific	A10036	IMF 1:500* 1:200**
Donkey anti-Mouse 647	Thermo Fisher Scientific	A31571	IMF 1:500*
Donkey anti-Goat 647	Thermo Fisher Scientific	A32849	IMF 1:500* 1:200**
Goat anti-Chicken 647	Thermo Fisher Scientific	A21449	IMF 1:500*

IHC, immunohistochemistry; IMF, immunofluorescence; WB, Western blot.
*Cultured neurons and mouse brain samples **Human brain samples

Supplementary Table 3. Primers used for qRT-PCR.

Target Genes	Forward (5´-3´)	Reverse (5´-3´)
PRKD1	CCCACGCTCTCTTTGTTCA	TCTTATGGTAATTCAGACCACACC
PRKD2	GGCTCAGGGCAGTTTGGAG	CAATGACCTTAACTGCCACGTC
Prkd1	CTGAGTTCTCCACCAGTGTCC	GATGGAGACTTTTGCTCAAAGC
Prkd2	GCTCATGTGAAGCAACTAGCC	GTCGTACAGGCCGTAGAAGC

Housekeeping Genes	Forward (5´-3´)	Reverse (5´-3´)
GAPDH	AGCCACATCGCTCAGACAC	CGCCCAATACGACCAAAT
β-*ACTIN*	CAGGCACCAGGGCGTG	GTGAGGATGCCTCTCTTGCTCT
β-*TUBULIN*	CTTTGTGGAATGGATCCCCA	GACTGCCATCTTGAGGCCA
18s rRNA	CTCAACACGGGAAACCTCAC	CGCTCCACCAACTAAGAACG
Gapdh	CTCCCACTCTTCCACCTTCG	CATACCAGGAAATGAGCTTGACAA
β-Actin	CTAAGGCCAACCGTGAAAAG	ACCAGAGGCATACAGGGACA
β-Tubulin	GACCTATCATGGGGACAGTGA	CGGCTCTGGGAACATAGTTT
