# Supplementary material for: Down-regulation of neuroprotective protein kinase D in Huntington´s disease
Source: Cell Death Dis. 2025 Jun 3;16(1):418. doi: 10.1038/s41419-025-07688-9 (PMC12134097; doi:10.1038/s41419-025-07688-9)
Supplement: Supplementary file 2 — Uncropped WB [file 41419_2025_7688_MOESM2_ESM.pdf]

A. Uncropped Western blots for Fig. 1C

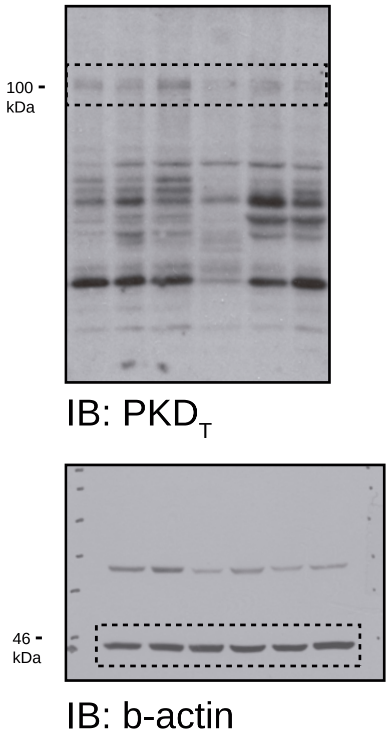

B. Uncropped Western blots for Fig. 2A

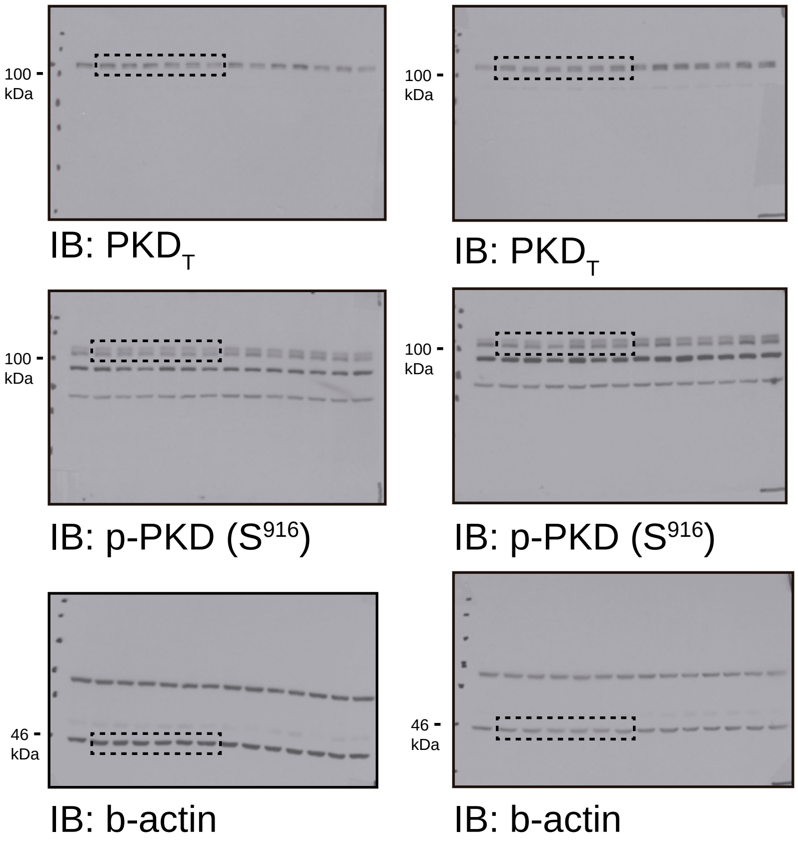

C. Uncropped Western blots for Fig. 2B

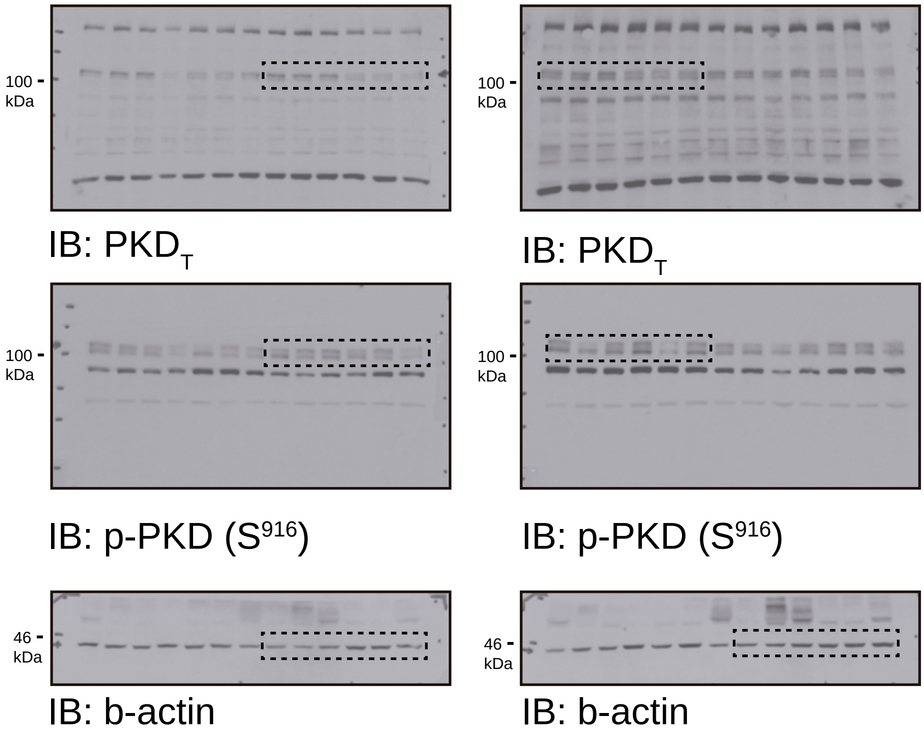

D. Uncropped Western blots for Fig. 4A

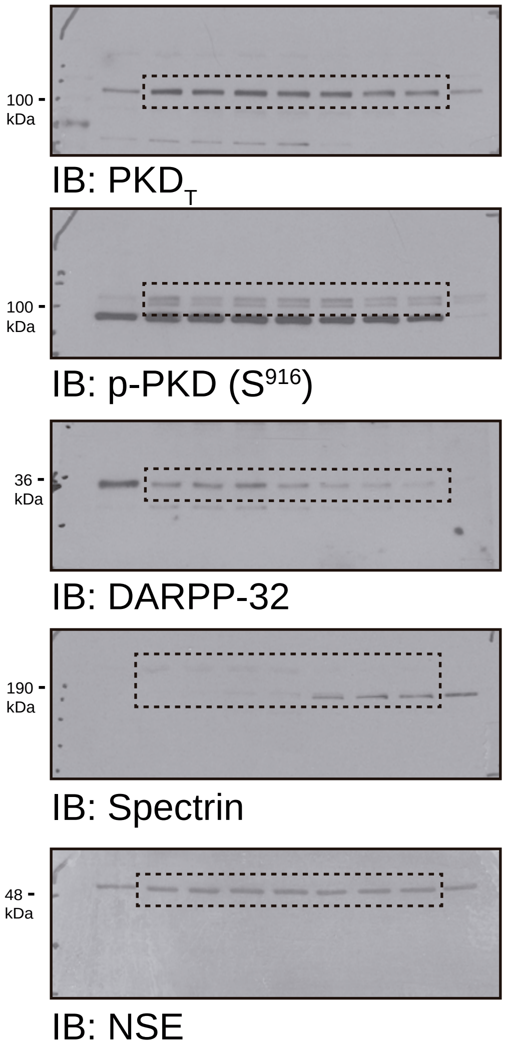

E. Uncropped Western blots for Fig. 4B

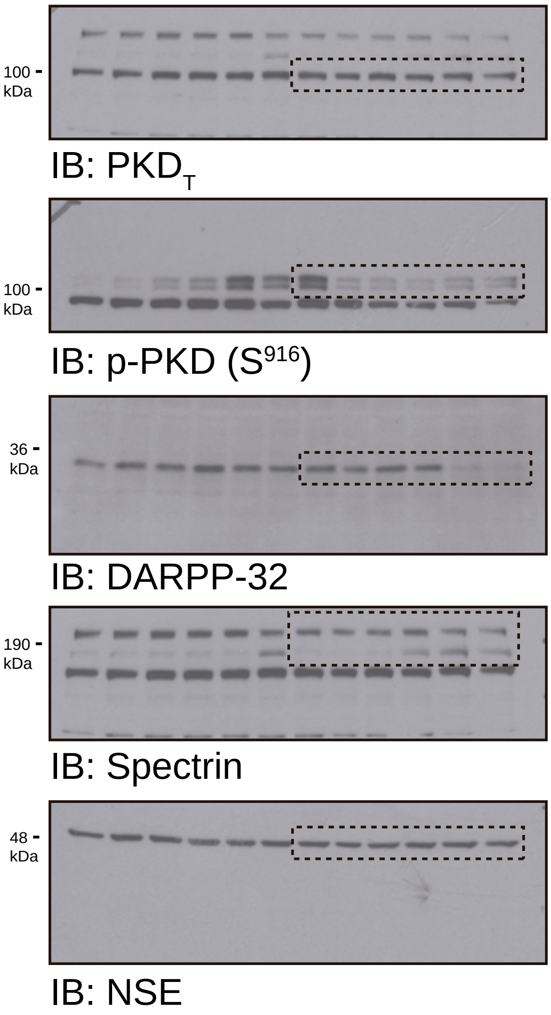

F. Uncropped Western blots for Fig. 4C

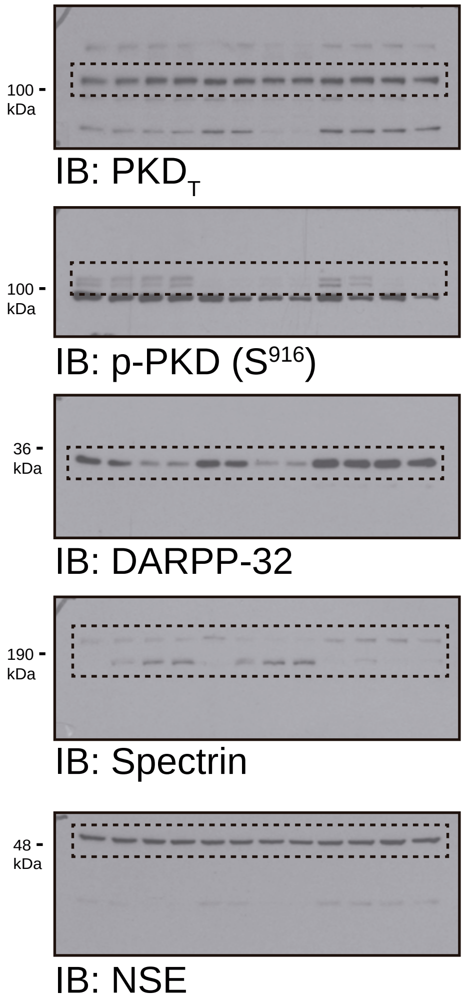

G. Uncropped Western blots for Fig. 4D

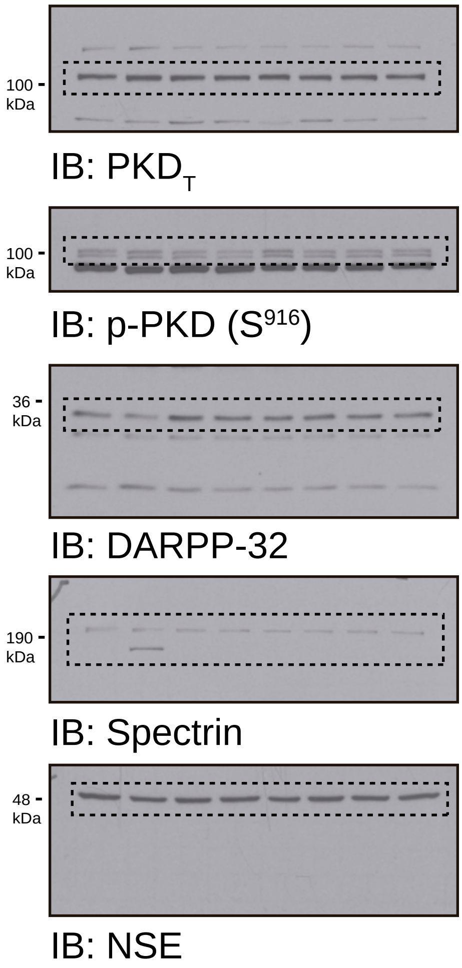

H. Uncropped Western blots for Fig. 7A

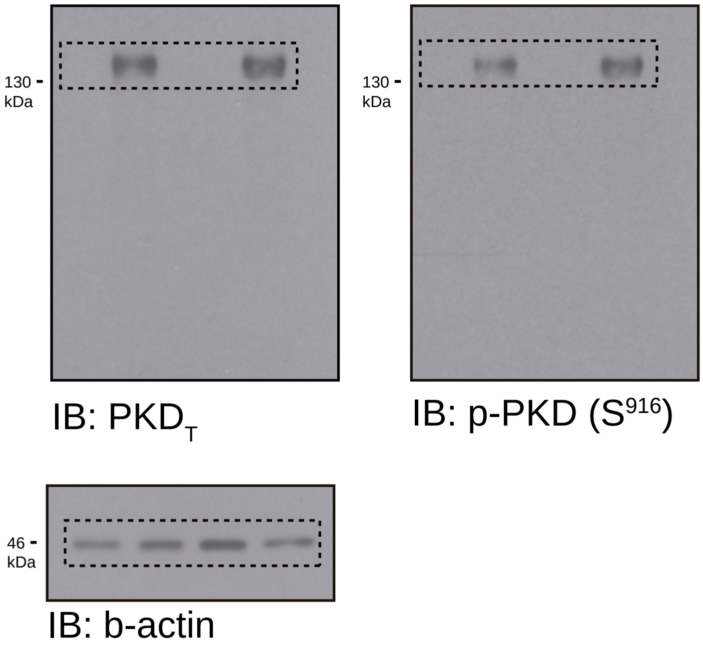

I. Uncropped Western blots for Supplementary Fig. 2

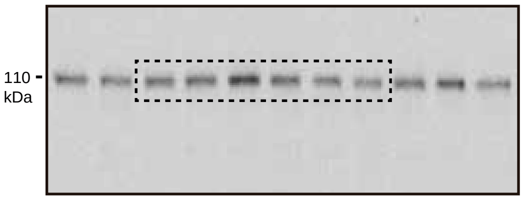

IB: PKD_T (Striatum)

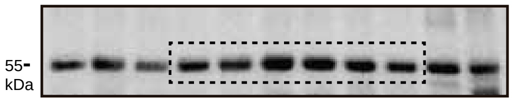

IB: α-tubulin (Striatum)

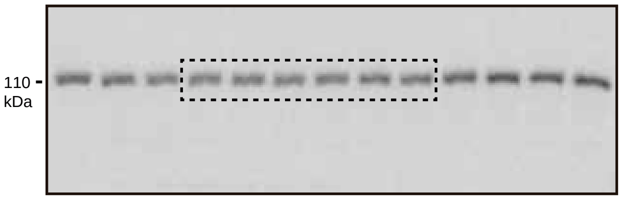

IB: PKD_T (Cortex)

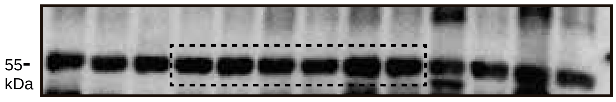

IB: α-tubulin (Cortex)

J. Uncropped Western blots for Supplementary Fig. 5

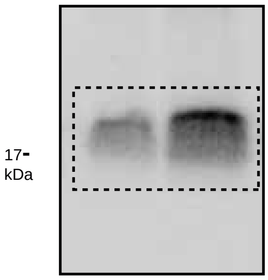

IB: Active Caspase-3

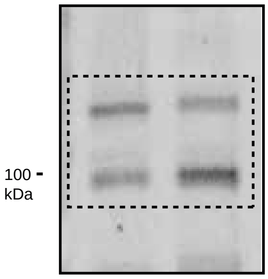

IB: PARP-1

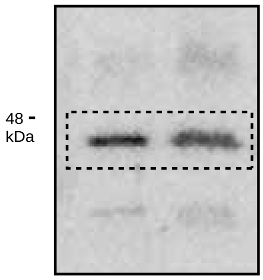

IB: NSE

K. Uncropped Western blots for Supplementary Fig. 7

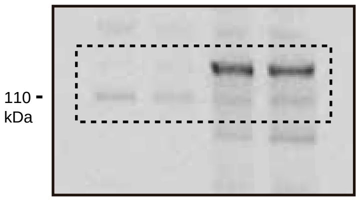

IB: PKD_T

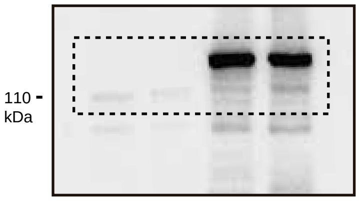

IB: p-PKD (S^{916})

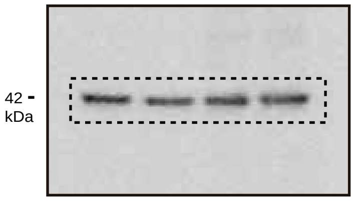

IB: NSE

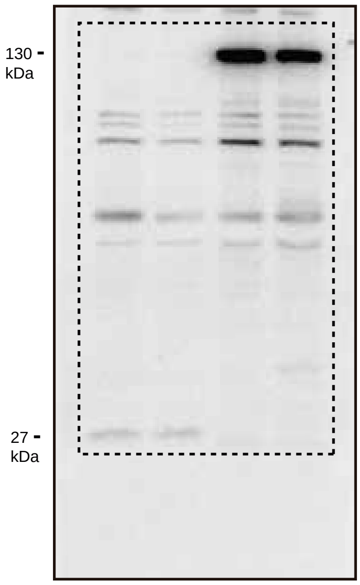

IB: GFP
